# Review of the species level taxonomy of the neotropical butterfly genus *Oenomaus* (Lycaenidae, Theclinae, Eumaeini)

**DOI:** 10.3897/zookeys.222.3375

**Published:** 2012-09-20

**Authors:** Christophe Faynel, Robert C. Busby, Robert K. Robbins

**Affiliations:** 1 16 rue des Aspres, F-34160 Montaud, France; 2 7 Countryside Way, Andover, MA 01810-6041 USA; 3 Department of Entomology, PO Box 37012, NHB Stop 105, Smithsonian Institution, Washington, DC 20013-7012 USA

**Keywords:** Annonaceae, Neotropics, *Porthecla*

## Abstract

Seven new species of the Neotropical hairstreak genus *Oenomaus* are described: *Oenomaus mancha* Busby & Faynel, **sp. n.** (type locality Ecuador); *Oenomaus gwenish* Robbins & Faynel, **sp. n.** (type locality Panama); *Oenomaus lea* Faynel & Robbins, **sp. n.** (type locality Ecuador); *Oenomaus myrteana* Busby, Robbins & Faynel, **sp. n.** (type locality Ecuador); *Oenomaus mentirosa* Faynel & Robbins, **sp. n.** (type locality Peru); *Oenomaus andi* Busby & Faynel, **sp. n.** (type locality Ecuador) and *Oenomaus moseri* Robbins & Faynel, **sp. n.** (type locality Brazil, Santa Catarina). For each new *Oenomaus* species, we present diagnostic characters and notes on its habitat and biology. We illustrate adults, genitalia, and distribution. New distributional and biological data are presented for 21 previously described *Oenomaus* species. *Oenomaus melleus guyanensis* Faynel, 2008 is treated as a new synonym of *Oenomaus melleus melleus* (Druce, 1907). Females are described and associated with males for ten species using a variety of factors, including mitochondrial COI DNA “barcode” sequences. We summarize the reasons why the number of recognized *Oenomaus* species has grown in the past decade from one species to 28 species. Finally, we overview the habitats that *Oenomaus* species occupy and note that the agricultural pest on Annonaceae, *Oenomaus ortygnus*, is the only *Oenomaus* species that regularly occurs in greatly disturbed habitats.

## Abstract (fr)

Sept nouvelles espèces appartenant au genre néotropical *Oenomaus* Hübner sont décrites : *Oenomaus mancha* Busby & Faynel, **sp. n.** (localité type : Equateur); *Oenomaus gwenish* Robbins & Faynel, **sp. n.** (localité type : Panama); *Oenomaus lea* Faynel & Robbins, **sp. n.** (localité type : Equateur); *Oenomaus myrteana* Busby, Robbins & Faynel, **sp. n.** (localité type : Equateur); *Oenomaus mentirosa* Faynel & Robbins, **sp. n.** (localité type : Pérou); *Oenomaus andi* Busby et Faynel, **sp. n.** (localité type : Equateur) et *Oenomaus moseri* Robbins & Faynel, **sp. n.** (localité type : Brésil, Santa Catarina). Pour chaque nouvelle espèce, les caractères diagnostiques sont présentés et sont accompagnés de remarques sur l’habitat et la biologie. Les faces dorsales et ventrales des adultes et les organes génitaux sont illustrés et des cartes de répartition sont données. Dans une deuxième partie, de nouvelles données sont présentées pour 21 espèces d’*Oenomaus* précédemment décrites. *Oenomaus melleus guyanensis* Faynel, 2008 est mis en synonymie avec *Oenomaus melleus melleus* (Druce, 1907). Les femelles sont décrites et associées avec des mâles en utilisant plusieurs moyens, y compris les séquences d’ADN mitochondrial COI. Les raisons pour lesquelles le nombre d’espèces d’*Oenomaus* décrites a fortement augmenté ces dernières années sont évoquées. Finalement, une vue d’ensemble des habitats occupés par les différentes espèces d’*Oenomaus* est présentée. *Oenomaus ortygnus*, qui est un parasite agricole connu sur Annonaceae, est la seule espèce d’*Oenomaus* qui se trouve régulièrement dans des habitats perturbés.

## Introduction

The widespread Neotropical hairstreak *Oenomaus ortygnus* (Cramer) is a pest of cultivated soursop (*Annona muricata* L.) and relatives (Annonaceae), and aspects of its biology have been documented for nearly a century (e.g., Dampf 1929; Fennah 1937; Ballou 1945; Guagliumi 1965, 1967; Araque 1967; d’Araújo e Silva et al. 1967–1968; Leal 1970; Kendall 1975; Domínguez 1978; Peña et al. 2002; Castañeda-Vildózola et al. 2011). In contrast, the taxonomy of the genus *Oenomaus* Hübner (Lycaenidae: Theclinae) was not addressed until recently. *Oenomaus* was considered to be a monotypic genus of uncertain affinity (Clench 1964) until Robbins (2004) listed 22 Neotropical species (18 undescribed). Shortly thereafter, Faynel (2006, 2008) and Faynel and Moser (2008) documented the substantive variation of male genitalic structures in *Oenomaus* and described 12 new species from male holotypes. However, associating females with the males was problematic for many of these species.

A close phylogenetic relationship between *Oenomaus* and *Porthecla* Robbins was suggested when Robbins and Duarte (2004) described the latter genus. However, the distinction between these two generahas been disputed because of different interpretations of male genitalic morphology, which has resulted in the uncertain generic placement for a few species (Faynel 2007; Faynel et al. 2011). The species level taxonomy of *Porthecla* has been treated (Faynel et al. 2011), but a similar overview for *Oenomaus* is lacking.

We present new species level taxonomic information for *Oenomaus* in this paper. We describe seven new *Oenomaus* species. Next, we update information on the distribution, habitat, variation, and biology of the 21 species that were previously described in or transferred to *Oenomaus* (Robbins 2004; Faynel 2007; Faynel et al. 2011). We also associate females with males for many species based on male-female pairs collected *in copula* or on similarity of ventral wing patterns, geographic distribution, and DNA ‘barcode’ sequences (the mitochondrial COI gene). The morphology of newly associated females is detailed. With the species level taxonomy of *Porthecla* recently reviewed (Faynel et al. 2011), the goal of this paper is do the same kind of review for *Oenomaus*. This information will serve as the foundation for a phylogenetic analysis of *Oenomaus* and *Porthecla*.

## Materials and methods

Genitalic terms follow those in Klots (1970), as modified for the Eumaeini in Robbins (1991). Wing veins are named following Comstock (1918), and wing cells are named by the veins that border them. Otherwise, morphological terms follow Snodgrass (1935). Abbreviations used repeatedly in the text are FW: forewing, HW: hindwing, D: dorsal, V: ventral and SD: standard deviation. Brazilian states are noted by their standard two letter abbreviations.

Illustrated adults of *Oenomaus* are noted in the material examined sections, and each genitalia drawing is of the adult illustrated. The structure of the male genitalia valvae in *Oenomaus* is complex, for which reason we present them in ventral, lateral, and dorsal views.

Biogeographical zones follow Brown (1982), who partitioned the forested continental Neotropics into the Transandean Region, Andean Region, Amazon Region, and Atlantic Region. Larval food plant nomenclature follows the Tropicos database of the Missouri Botanical Garden (http://www.Tropicos.org, accessed April 2012). Following Holdridge (1967), we classify lowland forests as humid/wet (> 200 cm annual precipitation) or dry/deciduous (100–200 cm annual precipitation). Many eumaeines display male territorial behavior on hilltops (Nicolay 1971; Alcock and O’Neill 1987; Prieto and Dahners 2006, 2009; Robbins et al. 2012). Males wait on hilltops for receptive females to fly through the territory and “defend” these areas by flying at other males that enter the territory. Recorded times from our fieldwork for hilltopping behavior are the standard time at that locality. Finally, traps baited with decaying fish attract some lycaenid species and not others. We note the gender for each species which has been collected using fish-baited traps.

The ventral wing pattern in *Oenomaus* is sexually monomorphic, so associating the sexes of species with distinct ventral wing patterns, such as *Oenomaus ortygnus*, is straightforward. However, a majority of *Oenomaus* species have a ventral wing pattern that is similar to that of *Oenomaus atena* (Hewitson). Among these, some can be associated because they have distinct ventral wing pattern elements, such as those of *Oenomaus isabellae* (Faynel 2008), or because a mating pair was collected *in copula*. In other cases, we associate females with males if at least three of the following four criteria are met: (1) females have a ventral wing pattern that is indistinguishable from that of males, (2) females have a geographic distribution that is similar to that of males, (3) both sexes are found in a locality where other species with the same wing pattern are unrecorded, and (4) divergence of DNA “barcode” sequences between the sexes is less than 2% (see next paragraph).

The mitochondrial COI gene sequence (commonly called a DNA “barcode”) has been useful, when combined with other characters, in distinguishing lepidopteran species in a single area (e.g., Hebert et al. 2004; Hajibabaei et al. 2006; Janzen et al. 2009). Because genitalic and wing pattern characters generally provide clear species boundaries in *Oenomaus*, our purpose in determining COI gene sequences was to aid in associating females with males, as noted.

We use the following acronyms for collections, following those for public institutions listed on the website hbs.bishopmuseum.org/codens/codens-inst.html (accessed April 2012):

AMNH American Museum of Natural History, New York, New York, USA.


ANSP Academy of Natural Sciences, Philadelphia, Pennsylvania, USA.


CF Private collection of Christophe Faynel, France.


CMNH Carnegie Museum of Natural History, Pittsburgh, Pennsylvania, USA.


DZUP Universidade Federal do Paraná, Curitiba, Paraná, Brazil.


FSMC Florida Museum of Natural History, Allyn Museum, University of Florida, Gainesville, Florida, USA.


JFLC Private collection of Jean François Le Crom, Bogotá, Colombia.


LYD Private collection of Louis and Yvan Diringer, France.


MC Private collection of Alfred Moser, São Leopoldo, Rio Grande do Sul, Brazil.


MNHN Muséum national d’Histoire naturelle, Paris, France.


MUSM Museo de Historia Natural, Universidad Nacional Mayor de San Marcos, Lima, Perú.


OSAC Oregon State University Corvallis, Oregon, USA.


PB Private collection of Pierre Boyer, Le Puy Sainte Réparade, France.


RCB Private collection of Robert C. Busby, Andover, Massachusetts, USA.


SMF Forschungsinstitut und Naturmuseum Senckenberg, Frankfurt-am-Main, Germany.


USNM Smithsonian Institution, Washington, DC USA.


## New species

**Figures 1–11.**
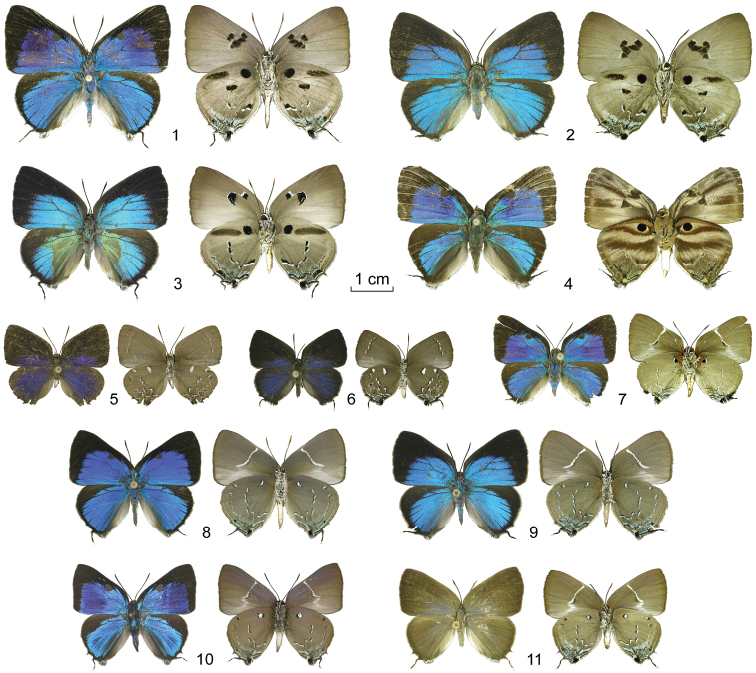
*Oenomaus*, new species: adults (dorsal surface at left, ventral surface at right). **1** ♂ *Oenomaus mancha* (holotype, Ecuador) **2** ♀ *Oenomaus mancha* (paratype, Ecuador) **3** ♀ *Oenomaus gwenish* (holotype, Panama) **4 ** ♂ *Oenomaus lea* (holotype, Peru) **5** ♂ *Oenomaus myrteana* (holotype, Ecuador) **6** ♀ *Oenomaus myrteana* (paratype, Ecuador) **7** ♂ *Oenomaus mentirosa* (holotype, Peru) **8** ♂ *Oenomaus andi* (holotype, Ecuador) **9** ♀ *Oenomaus andi* (paratype, Ecuador) **10** ♂ *Oenomaus moseri* (holotype, Brazil) **11** ♀ *Oenomaus moseri* (paratype, Brazil).

A distinguishing trait of *Oenomaus* and *Porthecla* among members of the *Panthiades* Section of the Eumaeini is the lack of an orange cubital spot (Robbins and Duarte 2004, Faynel et al. 2011). Of the seven new species described in this paper, six lack the spot while some specimens of the seventh species may have a vestigial remnant composed of a few orange scales (Figs 1–11). *Oenomaus* and *Porthecla* are distinguished from each other by shape of the valvae in lateral aspect, but interpretation of this morphology has varied for some species (Robbins and Duarte 2004, Faynel 2007, Faynel et al. 2011). However, six of the newly described species have non-triangular, bifurcate valvae in lateral aspect (Figs 20–25), which is characteristic of *Oenomaus*. The seventh species is known only from a female, but the similarities in its wing pattern (Figs 2–3) and genitalia (Figs 28–29) to two other *Oenomaus* species support its generic placement. For these reasons, the following new species are described in *Oenomaus*.

### Oenomaus mancha

Busby & Faynel sp. n.

urn:lsid:zoobank.org:act:4DF6102F-0A25-46B8-8F56-6E33D791A5F2

http://species-id.net/wiki/Oenomaus_mancha

Figs 1, 2 , 20 , 26 , 38 , 46

#### Type-locality.

Ecuador: Sucumbíos, 5 km Puerto Libre-La Bonita Road, 0°13.0'N, 77°29.3'W, 700 m. The road going west from Puerto Libre increases in elevation as the terrain becomes hillier. The collecting spot was in wet forest and was easily accessed by a muddy logging trail. Since 2005, logging has continued, leaving very few tall trees in the once beautiful forest.

#### Type-specimen.

**Holotype** ♂ (Fig. 1) labeled as “ECUADOR: Sucumbios / 5 km Puerto Libre-La Bonita Road / 0°13.0'N, 77°29.3'W, 700 m / 23 February 2005 / Robert C. Busby, leg.” [rectangular, white, printed], “11:00 hrs / 5 m” [rectangular, white, handwritten, blue ink], “GENITALIA No. / 2011: 419♂ / C. FAYNEL” [rectangular, green, printed] “Holotype ♂ / *Oenomaus mancha* / Busby & Faynel, 2012” [rectangular, red, printed]. Deposited in USNM.

**Paratypes: Ecuador**. **2** ♂: Napo, 14 km Tena-Puyo Road, 1°06.7'S, 77°46.9' W, 600 m, 24.IX.2011, (Apuya) Robert C. Busby leg. (RCB); Napo, Pimpilala, [ GPS data : 1°04.6 S, 77°56.2'W ], 600–700 m, Euclides Aldaz leg., XII.2003, gen. prep. CF n°290 (PB); **10**♀: Napo, 28 km Tena-Puyo Road, 1°11.3'S, 77°49.9'W, 800 m, VIII.2006 (El Capricho) I. Aldas & R. C. Busby leg. (RCB); Napo, 12 km Tena-Puyo Road, 1°05.3'S, 77°47.4' W, 600 m, 28.VIII.2009, (Finca San Carlo) D. H. Ahrenholz, R. C. Busby, leg. (RCB); Napo, 14 km Tena-Puyo Road, 1°06.7'S, 77°46.9'W 600 m, VIII.2005, (Apuya) I. Aldas & R. C. Busby leg. (RCB) ; Napo, 14 km Tena-Puyo Road, 1°06.7'S, 77°46.9'W, 600 m, 17.X.2010, (Apuya) I. Aldas & R. C. Busby leg. (RCB); Napo, 14 km Tena-Puyo Road, 1°06.7'S, 77°46.9'W, 600 m, 22.X.2010, (Apuya) I. Aldas & R. C. Busby leg. (RCB); Pastaza Province, 32 km S. of Puyo, 1000 m, 21–23.X.1995 Robert C. Busby leg. (RCB); Pastaza Province, 45 km Puyo-Arajuno Rd, 1000 m, 15.IX.1999, Robert C. Busby leg., gen. prep. CF n°420 (RCB); Pastaza Province, 45 km Puyo-Arajuno Rd, 1000 m, 26.IX.1999, Robert C. Busby leg., gen. prep. CF n°421 (RCB); Pastaza Province, 45 km Puyo-Arajuno Rd, 1000 m, 26.IX.1999, Robert C. Busby leg. (RCB); Pastaza, Puyo, 1000 m, 14.X.1989, D.H. Ahrenholz leg., gen. prep. CF n°407 (USNM ENT 00180037) (Fig. 2).

#### Description, diagnosis and recognition as a distinct species.

Male FW length: 20.8 mm (SD = 1.9, *N* = 2). Female FW length: 19.4 mm (SD = 0.5, *N* = 3). Wing pattern (Figs 1, 2) and genitalia (Figs 20, 26) illustrated. *Oenomaus mancha*, *Oenomaus ortygnus*,and *Oenomaus gwenish* (named below) share a unique ventral wing pattern in which the VFW postmedian line (displaced basally, but by tradition still called the postmedian line) is composed of “disjointed” large black spots on a gray ground color (Fig. 3 for *Oenomaus gwenish* and Figs 2, 4 for *Oenomaus ortygnus* in Faynel 2006). *Oenomaus mancha* differs from *Oenomaus ortygnus*by (1) a black patch in the distal part of the VHW cell Sc+R1-Rs, elongated basally, (2) no black mark in VFW cell Costa-Sc, and (3) a black band crossing the VFW discal cell. In addition, females of *Oenomaus mancha* are a brighter blue dorsally, while the blue on the DFW of males is somewhat less expansive with the scent pad not completely encircled by blue scales as in *Oenomaus ortygnus*. Male and female genitalia of *Oenomaus mancha* and *Oenomaus ortygnus* also differ (Figs 25, 28 for *Oenomaus ortygnus* in Faynel 2006). In particular, the dorsal part of the valvae of the male genitalia in lateral aspect is shorter and has a more sharply tapered posterior end in ventral view. In the female, the bifid posterior end of the lamella postvaginalis is less marked and the anterior end of the ductus bursae is curved more sharply. One paratype from Ecuador has been barcoded (CF-LYC-190), and its sequence is 3.5% divergent from the sequences of two males of *Oenomaus ortygnus* (CF-LYC-147 from Peru and CF-LYC-146 from Mexico, see Table 1) while the two *Oenomaus ortygnus* sequences differ by only 1.5%. *Oenomaus ortygnus* and *Oenomaus mancha* are sympatric in eastern Ecuador in Napo Province at approximately 450 m.

**Figures 12–19.**
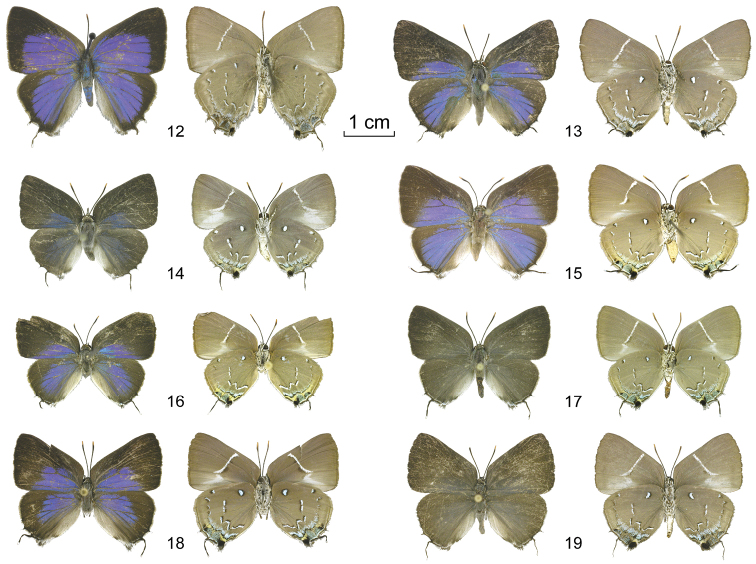
*Oenomaus*, newly associated females: adults (dorsal surface at left, ventral surface at right). **12** *Oenomaus geba* (Brazil) **13** *Oenomaus magnus* (French Guiana) **14** *Oenomaus brulei* (French Guiana) **15** *Oenomaus gaia* (Panama) **16** *Oenomaus cyanovenata* (Costa Rica) **17** *Oenomaus cyanovenata* (French Guiana) **18** *Oenomaus taua* (Panama) **19** *Oenomaus taua* (Ecuador).

**Figures 20–25.**
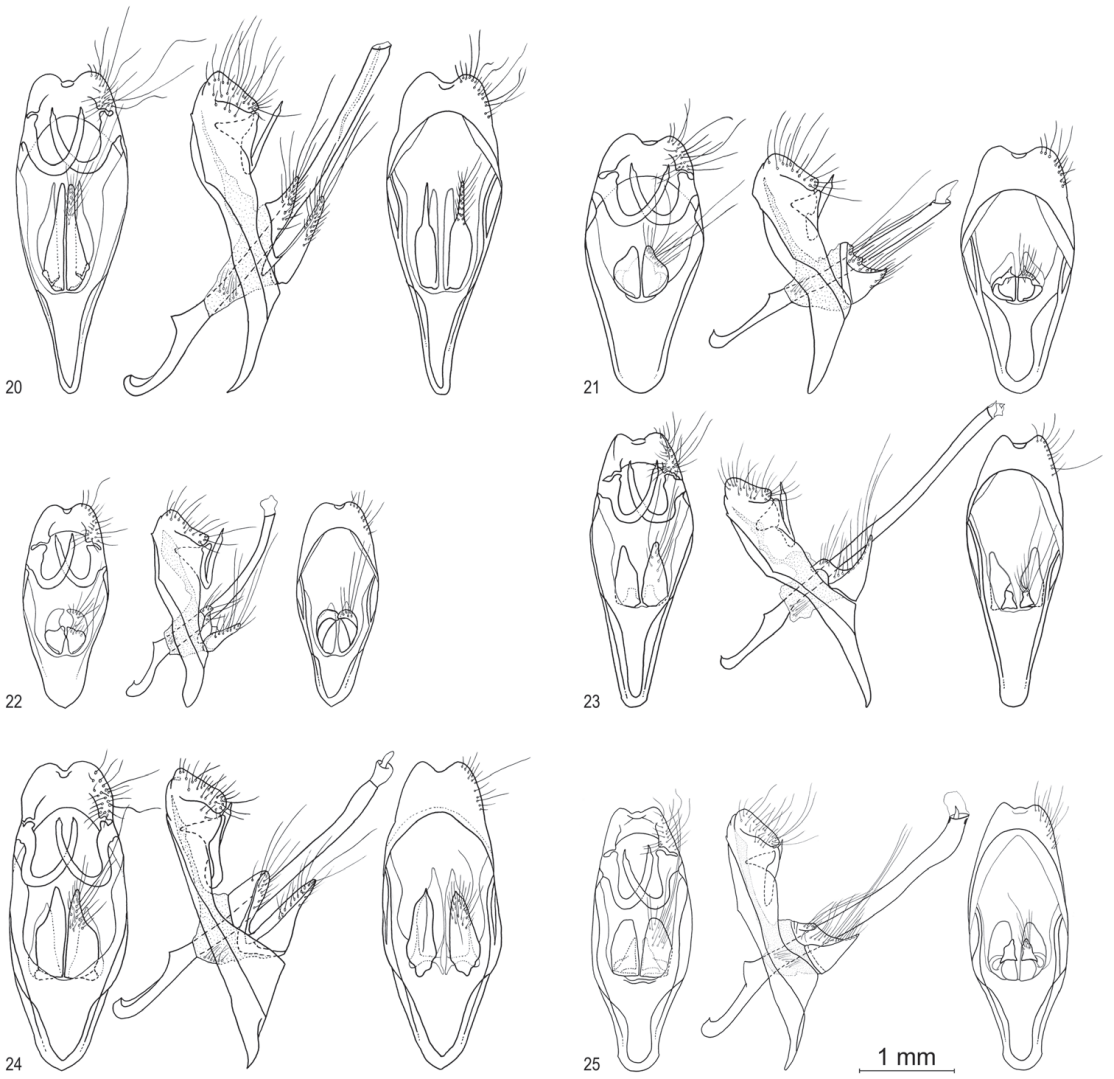
*Oenomaus* male genitalia: ventral view at left (setae drawn only on the right side and penis removed), left lateral view in the middle (with the penis and valvae displaced ~45° to make them more visible), and dorsal view at right (with setae drawn only on the right side and penis removed). **20**  *Oenomaus mancha* (holotype, Ecuador) **21** *Oenomaus lea* (holotype, Peru) **22** *Oenomaus myrteana* (holotype, Ecuador) **23 ***Oenomaus mentirosa* (holotype, Peru) **24** *Oenomaus andi* (holotype, Ecuador) **25** *Oenomaus moseri* (holotype, Brazil).

**Figures 26–37.**
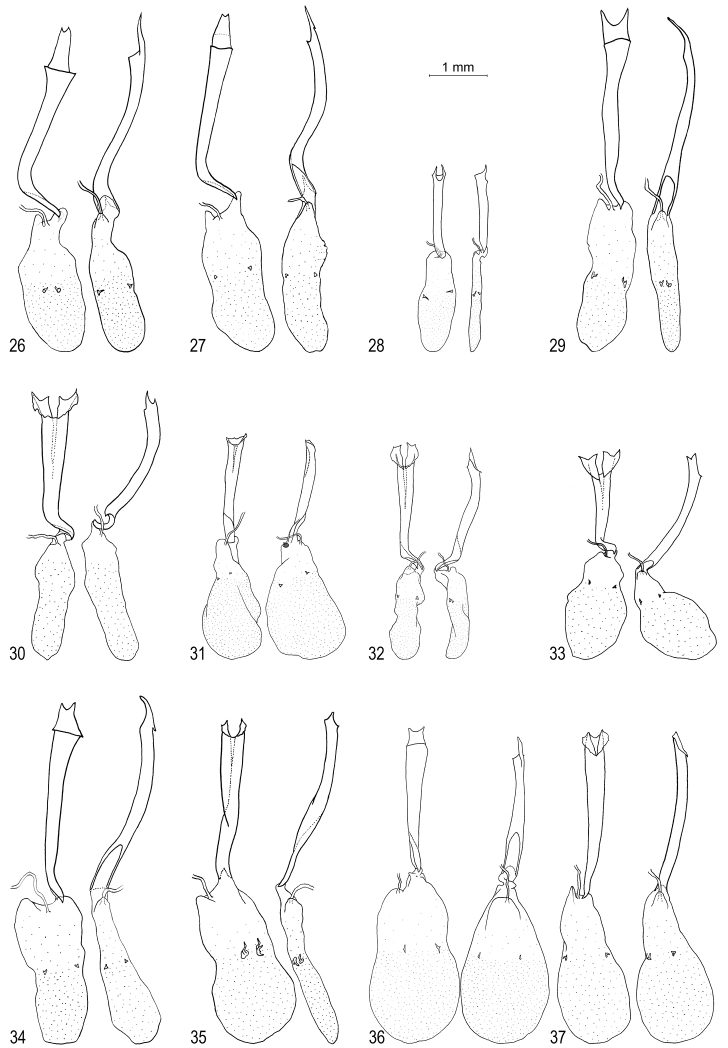
*Oenomaus* female genitalia: ventral view at left, lateral view on right. **26** *Oenomaus mancha* (paratype, Ecuador) **27** *Oenomaus gwenish* (holotype, Panama) **28** *Oenomaus myrteana* (paratype, Ecuador) **29** *Oenomaus andi* (paratype, Ecuador) **30** *Oenomaus moseri* (paratype, Brazil) **31** *Oenomaus brulei* (French Guiana) **32** *Oenomaus cyanovenata* (French Guiana) **33** *Oenomaus cyanovenata* (Costa Rica) **34** *Oenomaus gaia* (Panama) **35** *Oenomaus geba* (Brazil) **36** *Oenomaus magnus* (French Guiana) **37** *Oenomaus taua* (Panama).

**Table 1.** Comparison of inter- and intraspecific divergences (in % rounded to the nearest tenth) for the DNA “barcodes” of 19 *Oenomaus* male species obtained with BOLD (noted as -- when not available). Number of males examined in brackets.

	***Oenomaus ambiguus***	***Oenomaus atena***	***Oenomaus atesa***	***Oenomaus brulei***	***Oenomaus cortica***	***Oenomaus curiosa***	***Oenomaus cyanovenata***	***Oenomaus gaia***	***Oenomaus isabellae***	***Oenomaus jauffreti***	***Oenomaus lea***	***Oenomaus magnus***	***Oenomaus mancha***	***Oenomaus morroensis***	***Oenomaus moseri***	***Oenomaus nigra***	***Oenomaus ortygnus***	***Oenomaus poirieri***	***Oenomaus taua***
*Oenomaus ambiguus* (3)	0.2																		
*Oenomaus atena* (1)	6.9	--																	
*Oenomaus atesa* (1)	7.2	7.0	--																
*Oenomaus brulei* (2)	6.2	6.2	7.4	0.3															
*Oenomaus cortica* (3)	2.8	5.5	7.5	5.9	0.5														
*Oenomaus curiosa* (4)	7.9	6.5	5.3	6.9	7.4	0.6													
*Oenomaus cyanovenata* (4)	7.1	5.4	7.2	7.3	5.8	6.4	0												
*Oenomaus gaia* (3)	2.4	5.3	7.0	5.6	1.0	7.2	5.9	0.7											
*Oenomaus isabellae* (1)	6.2	6.7	7.6	7.3	5.8	7.9	6.1	5.6	--										
*Oenomaus jauffreti* (4)	7.0	6.5	7.0	6.2	6.4	7.6	6.9	6.1	4.9	1.6									
*Oenomaus lea* (1)	6.9	4.7	6.7	6.3	6.1	6.2	5.9	5.9	7.3	5.8	--								
*Oenomaus magnus* (1)	7.9	6.9	7.6	7.6	7.0	7.5	6.9	6.8	4.5	5.9	7.7	--							
*Oenomaus mancha* (1)	5.2	5.2	5.7	5.7	4.9	5.9	5.6	4.3	4.9	5.4	5.6	6.4	--						
*Oenomaus morroensis* (1)	2.3	4.8	7.1	4.8	0.9	7.1	5.4	0.9	5.7	6.3	5.7	6.8	5	--					
*Oenomaus moseri* (2)	5.7	5.9	6.7	6.9	5.7	6.9	4.0	5.2	5.6	5.8	5.8	6.6	4.9	5.1	0				
*Oenomaus nigra* (1)	7.7	6.7	5.7	8.4	7.8	5.9	7.6	7.2	7.8	6.9	5.9	7.2	6.4	7.7	7	--			
*Oenomaus ortygnus* (2)	6.7	5.7	6.6	7.1	6.3	6.4	6.3	6.1	6.3	6.3	6.3	6.2	3.5	6.2	5.4	6.6	1.5		
*Oenomaus poirieri* (1)	7.7	6.9	7.5	7.2	7.5	8.0	6.6	7.0	5.2	4.6	7.5	6.2	6.1	6.9	5.3	7.6	6.2	--	
*Oenomaus taua* (2)	5.9	5.2	5.4	6.1	5.3	6.0	5.1	4.9	5.2	4.7	4.3	5.9	4.1	5.4	4.8	6.1	4.9	5.6	0

**Table 2.** Species of *Oenomaus* sampled with BOLD (project NLYCA), with sample identifications, localities and GenBank accession numbers.

**Species**	**Sample ID**	**Locality**	**GenBank Accession Numbers**
*Oenomaus ambiguus* Faynel, 2008	CF-LYC-025	Peru	HQ966548
*Oenomaus ambiguus* Faynel, 2008	CF-LYC-183	Peru	JX458731
*Oenomaus ambiguus* Faynel, 2008	CF-LYC-189	Peru	JX458734
*Oenomaus atena* (Hewitson, 1867)	CF-LYC-084	Peru	HQ966592
*Oenomaus atesa* (Hewitson, 1867)	CF-LYC-003	French Guiana	HQ966543
*Oenomaus brulei* Faynel, 2008	CF-LYC-033	French Guiana	HQ966552
*Oenomaus brulei* Faynel, 2008	CF-LYC-035	French Guiana	HQ966554
*Oenomaus cortica* (D'Abrera, 1995)	CF-LYC-051	Brazil	HQ966565
*Oenomaus cortica* (D'Abrera, 1995)	CF-LYC-052	Brazil	HQ966566
*Oenomaus cortica* (D'Abrera, 1995)	CF-LYC-188	Peru	JX458722
*Oenomaus curiosa* Faynel & Moser, 2008	CF-LYC-036	French Guiana	HQ966555
*Oenomaus curiosa* Faynel & Moser, 2008	CF-LYC-037	French Guiana	HQ966556
*Oenomaus curiosa* Faynel & Moser, 2008	CF-LYC-016	Peru	JX458726
*Oenomaus curiosa* Faynel & Moser, 2008	CF-LYC-184	Peru	JX458730
*Oenomaus cyanovenata* (D'Abrera, 1995)	CF-LYC-049	Brazil	HQ966564
*Oenomaus cyanovenata* (D'Abrera, 1995)	CF-LYC-048	Brazil	HQ966563
*Oenomaus cyanovenata* (D'Abrera, 1995)	CF-LYC-047	French Guiana	JX458737
*Oenomaus cyanovenata* (D'Abrera, 1995)	CF-LYC-182	Peru	JX458728
*Oenomaus gaia* Faynel, 2008	CF-LYC-024	Peru	JX458720
*Oenomaus gaia* Faynel, 2008	CF-LYC-023	French Guiana	JX458719
*Oenomaus gaia* Faynel, 2008	CF-LYC-187	Peru	JX458721
*Oenomaus isabellae* Faynel, 2006	CF-LYC-006	Brazil	HQ966545
*Oenomaus jauffreti* Faynel & Moser, 2008	CF-LYC-030	Brazil	JX458724
*Oenomaus jauffreti* Faynel & Moser, 2008	CF-LYC-029	French Guiana	HQ966549
*Oenomaus jauffreti* Faynel & Moser, 2008	CF-LYC-028	Brazil	JX458727
*Oenomaus jauffreti* Faynel & Moser, 2008	CF-LYC-186	Peru	JX458732
*Oenomaus lea* Faynel & Robbins, 2012	CF-LYC-005	Peru	HQ966544
*Oenomaus magnus* Faynel & Moser, 2008	CF-LYC-020	Peru	HQ966547
*Oenomaus mancha* Busby & Faynel, 2012	CF-LYC-190	Ecuador	JX458723
*Oenomaus morroensis* Faynel & Moser, 2008	CF-LYC-015	Brazil	JX458736
*Oenomaus moseri* Robbins & Faynel, 2012	CF-LYC-012	Brazil	JX458735
*Oenomaus moseri* Robbins & Faynel, 2012	CF-LYC-063	Brazil	HQ966576
*Oenomaus nigra* Faynel & Moser, 2008	CF-LYC-148	Peru	JX458729
*Oenomaus ortygnus* (Cramer, 1779)	CF-LYC-146	Mexico	JX458738
*Oenomaus ortygnus* (Cramer, 1779)	CF-LYC-147	Peru	JX458733
*Oenomaus poirieri* Faynel, 2008	CF-LYC-011	French Guiana	JX458725
*Oenomaus taua* Faynel & Moser, 2008	CF-LYC-085	Peru	HQ966593
*Oenomaus taua* Faynel & Moser, 2008	CF-LYC-185	Peru	JX458739

#### Etymology.

The name of this species is derived from the Spanish word “mancha”, which means spot, referring to the very distinctive, elongated black spot in VHW cell Sc+R1-Rs. The name is a feminine noun in apposition.

#### Habitat and distribution.

*Oenomaus mancha* occurs widely in wet forest in eastern Ecuador at elevations ranging from 400 to 1100 m (Fig. 46). Although it is sympatric with *Oenomaus ortygnus* in wet forest, it does not occur in the highly disturbed habitats in which *Oenomaus ortygnus* sometimes occurs. It is yet an open question whether *Oenomaus mancha* is a lowland or lower montane species.

**Figures 38–45.**
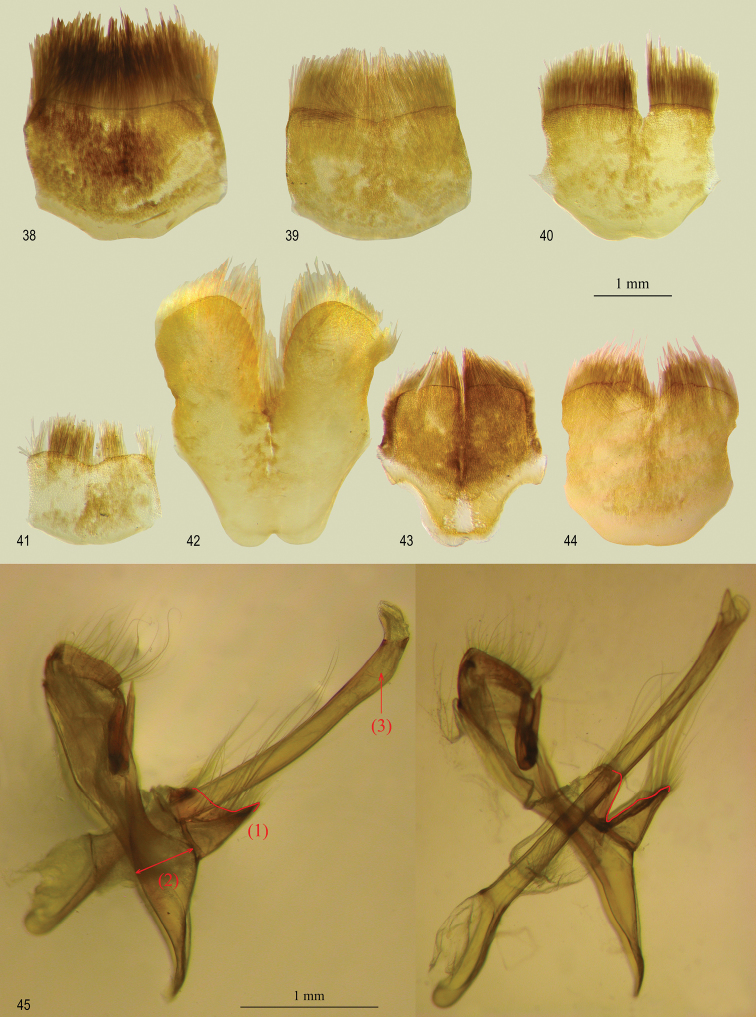
*Oenomaus*, eighth abdominal tergum: ventral view, anterior edge at bottom. **38** ♂ *Oenomaus mancha* (holotype, Ecuador) **39** ♂ *Oenomaus lea* (holotype, Peru) **40** ♂ *Oenomaus mentirosa* (holotype, Peru) **41** ♂ *Oenomaus myrteana* (holotype, Ecuador) **42** ♂ *Oenomaus andi* (holotype, Ecuador) **43** ♀ *Oenomaus andi* (paratype, Ecuador) **44** ♂ *Oenomaus moseri* (holotype, Brazil) **45** Male genitalia in lateral view: *Oenomaus moseri* (left) and *Oenomaus morroensis* holotypes. Diagnostic characters (1), (2) and (3) are explained in the text.

**Figures 46–47.**
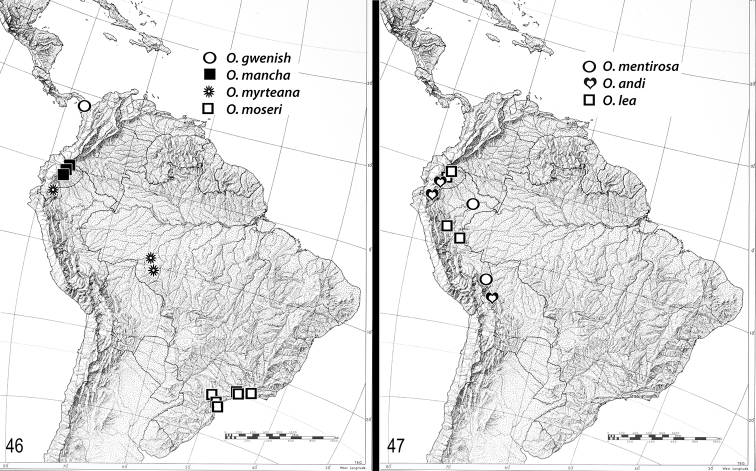
Distributions. **46** *Oenomaus mancha*, *Oenomaus gwenish*, *Oenomaus myrteana*, *Oenomaus moseri* **47** *Oenomaus andi*, *Oenomaus lea*, *Oenomaus mentirosa*.

#### Behavior.

The holotype male was landed on a leaf about 5 m above the ground at 11:00 hours. Males and females are attracted to traps baited with rotting fish (vouchers in RCB).

### Oenomaus gwenish

Robbins & Faynel sp. n.

urn:lsid:zoobank.org:act:D35807B0-C3C3-4384-BB59-E294B3E06F97

http://species-id.net/wiki/Oenomaus_gwenish

Figs 3 , 27 , 46

#### Type-locality.

Panama: Darién, Serranía de Pirre, Cana, 7°55'57"N, 77°42'58"W, 1000 m. Serranía de Pirre at 1000 m was uncut wet lower montane forest in 1984. The only disturbance was a defunct gold mine camp and associated dirt runway at Cana.

#### Type-specimen.

**Holotype** ♀ (Fig. 3) labeled as “PANAMA: Darien: / Serrania de Pirre: / Cana: 1,000 m / 5 January 1984 / Leg. G.B. Small” [rectangular, white, printed and handwritten], “GENITALIA NO. / 2011: 406♀ / C. FAYNEL” [rectangular, green, printed] “Holotype ♀ / *Oenomaus gwenish* / Robbins & Faynel, 2012” [rectangular, red, printed]. Deposited in USNM.

#### Description, diagnosis and recognition as a distinct species.

Female FW length: 20 mm (*N* = 1). Wing pattern (Fig. 3) and genitalia (Fig. 27) illustrated. The wing patterns of *Oenomaus gwenish* and *Oenomaus mancha* are distinguished from that of *Oenomaus ortygnus*by the black patch in the distal part of the VHW cell Sc+R1-Rs and by the absence of a black mark in VFW cell Costa-Sc. However, the ventral wing pattern of *Oenomaus gwenish* differs from that of *Oenomaus mancha* by (1) the lack of a black band crossing the VFW discal cell, (2) the absence of a black spot in VFW cell M3-Cu1, and (3) the presence of two black spots of equal size along VHW veins mdc and ldc instead of a single large black spot at vein mdc with no mark or a faint vestigial mark at vein ldc. Thefemalegenitalia of *Oenomaus gwenish* (Fig. 27) are similar to those of *Oenomaus mancha*.

We hesitated to describe this species because we cannot assess its intraspecific variation. However, the series of 10 females of *Oenomaus mancha* show little variation in the traits that distinguish them from the holotype of *Oenomaus gwenish*. For this reason, a hypothesis of specific distinctness is better supported than a hypothesis of geographical variation.

#### Etymology.

The holotype of *Oenomaus gwenish* is a unique and distinctive female, for which reason it gives us great pleasure to name this species for entomologist Dr. Jennifer (Gwen) Shlichta. The name is a feminine noun in apposition.

#### Habitat and distribution.

*Oenomaus gwenish* is probably a lower montane species, so far known only from wet forest at 1000 m elevation in Darién, Panama (Fig. 46). While *Oenomaus gwenish* and *Oenomaus ortygnus* are both known from Panama, we do not know if they are sympatric.

### Oenomaus lea

Faynel & Robbins sp. n.

urn:lsid:zoobank.org:act:A8315354-6FFF-4C71-AEE5-92925AE8833B

http://species-id.net/wiki/Oenomaus_lea

Figs 4 , 21 , 39 , 47

#### Type-locality.

Ecuador: Napo, Misahuallí Rd, Latas Grande, 7.7 km E Puerto Napo, 1°02.0'S, 77°44.1'W, 470 m. The holotype was collected along the road from Tena to Misahuallí, which in 1991 was a patchwork of “fincas” and remnant second growth forest.

#### Type-specimen.

**Holotype** ♂ (Fig. 4) labeled as “ECUADOR Napo / Misahualli Rd. 470m / Latas Grande / 9 Nov.’ 91 / S. S. Nicolay” [rectangular, white, printed and handwritten], “USNM ENT 00180040” [rectangular, white, printed], “GENITALIA NO. / 2011: 408♂ / C. FAYNEL” [rectangular, green, printed] “Holotype ♂ / *Oenomaus lea* / Faynel & Robbins, 2012” [rectangular, red, printed]. Deposited in USNM.

**Paratypes: Ecuador.** **1** ♂: La Merced on Río Pastaza below Baños, Alt. 4000 ft. [= 1220 m], W. J. Coxey, III.1930, A.N.S. Lot 217, genitalia NO. 1992: 12 ♂ R.K. Robbins (ANSP). **Peru**. **3** ♂: UC, Pucallpa, 200 m, X.2007, Michael Büche leg. (CF); LO, Contamana, Río Ucayali, 300 m, 7°19'S, 74°48'W, IX.2010, leg J. Ramírez (LYD); SM, Juanjui, upper Huallaga River, IX. 1934, collector G. Klug, collection E.I. Huntington NO. 1055 (AMNH).

#### Description, diagnosis and recognition as a distinct species.

Male FW length: 18.2 mm (SD = 0.8, *N* = 4). Wing pattern (Fig. 4) and genitalia (Fig. 21) illustrated. *Oenomaus lea* and *Oenomaus atesa* (Hewitson, 1867)are the only two Eumaeini sharing the striking underside wing pattern with two transverse brown bands on the VHW. However, males of *Oenomaus lea* differ from males of *Oenomaus atesa* (Figs 5, 6 in Faynel 2006) by (1) a greater expanse of the DFW blue, especially in the area from vein R3 to vein Cu1, (2) two brown patches on the VFW instead of a single median brown band; one patch is located along the costa, and the other is triangular and situated in the basal part of cell Cu1-Cu2, and (3) a lighter dorsal blue color with a different hue of blue along the HW veins M2, M3, Cu1 and Cu2. Moreover, the black spot in VHW cell Cu1-Cu2 is usually more apparent in *Oenomaus atesa* than in *Oenomaus lea*. Males of *Oenomaus lea* alsodiffer genitalically from those of *Oenomaus atesa* (Fig. 26 in Faynel 2006) by (1) a longer and wider saccus, (2) the dorsal part of the valvae in lateral aspect shorter than the ventral part, and not pointed at the posterior end, (3) a straight penis in lateral view, and (4) no tooth at the end of the penis. The eighth tergum shows no difference from that of *Oenomaus atesa*. Lastly, the divergence of “barcode” DNA sequence data between *Oenomaus lea* (CF-LYC-005) and *Oenomaus atesa* (CF-LYC-003) is more than 6% (Table 1). The female of *Oenomaus lea* is unknown.

#### Etymology.

This species is named for Léa Faynel, daughter of Christophe Faynel. The name is a feminine noun in apposition.

#### Habitat and distribution.

*Oenomaus lea* occurs in wet lowland forest up to 1200 m elevation in eastern Ecuador and eastern Peru (Fig. 47).

### Oenomaus myrteana

Busby, Robbins & Faynel sp. n.

urn:lsid:zoobank.org:act:B8EBB664-61DA-4178-91C8-52D2D9BFF0AC

http://species-id.net/wiki/Oenomaus_myrteana

Figs 5, 6 , 22 , 28 , 41 , 46

#### Type-locality.

Ecuador: Morona Santiago, Santiago (Hill North of Town), 3°02.3'S, 78°00.3'W, 350 m. The holotype was collected in wet secondary forest in the low hills on the north edge of Santiago.

#### Type-specimen.

**Holotype** ♂ (Fig. 5) labeled as “ECUADOR: Morona Santiago / Santiago (Hill North of Town) / 3°02.3'S, 78°00.3'W 350 m / 20 September 2004 / Robert C. Busby, leg.” [rectangular, white, printed], “GENITALIA NO. / 2003: 35♂ / R.K. ROBBINS” [rectangular, green, printed] “Holotype ♂ / *Oenomaus myrteana* / Busby, Robbins & Faynel, 2012” [rectangular, red, printed]. Deposited in USNM.

**Paratypes: Ecuador.** **1** ♂: Morona Santiago, Santiago (Hill North of Town), 3°2.3'S, 78°0.3'W, 350 m, 20.IX.2006, Robert C. Busby leg. (RCB) ; **1**♀: Morona-Santiago Province, 1.8 km Santiago-Puerto Morona Rd., 3°2.4'S, 77°59.7'W, 300–350 m, 20.IX.2006, D.H. Ahrenholz & Robert C. Busby leg., gen. prep. CF n°415 (RCB) (Fig. 6). **Brazil**. **2** ♂: RO, Cacaulândia, 1–5.IX.1997, E. Furtado & A. Moser leg., gen. prep. CF n°442 (MC 250); RO, Candeias do Jamari, Rio Preto, 27–31.VIII.1997, E. Furtado & A. Moser leg., gen. prep. CF n°443 (MC 251).

#### Description, diagnosis and recognition as a distinct species.

Male FW length: 12.8 mm (SD = 0.1, *N* = 3). Female FW length: 12.1 mm (*N* = 1). Wing pattern (Figs 5, 6) and genitalia (Figs 22, 28) illustrated. *Oenomaus myrteana* has a conspicuous round white spot in VHW cell Sc+R1-Rs, which is similar to those species of *Oenomaus* with a ventral wing patterns similar to that of *Oenomaus atena*. However, *Oenomaus myrteana* lacks the inclined white median line of the VFW, which is characteristic of species with the *Oenomaus atena* wing pattern. Instead, *Oenomaus myrteana* has a vertical, distally displaced postmedian line of white dashes, inwardly bordered by black. This character appears to be unique among *Oenomaus* species. In addition, *Oenomaus myrteana* may have a few red-orange scales in VHW cell Cu1-Cu2. This red-orange cubital spot is uniformly lacking in other *Oenomaus* and *Porthecla*. The male genitalia of *Oenomaus myrteana* are very similar to those of *Oenomaus nigra*, which has an “*atena*-like” ventral wing pattern. The female genitalia of *Oenomaus myrteana* are similar to those *Oenomaus* that have a bifid posterior end of the ductus bursae and a signa with a two pointed spine in the middle of the corpus bursae.

The ventral wing pattern of *Oenomaus myrteana* is superficially similar to those of *Enos myrtea* (Hewitson) and *Allosmaitia myrtusa* (Hewitson), but in these genera, males lack a scent pad on the DFW. The genitalia of *Oenomaus myrteana*, as noted, are typical of *Oenomaus*.

#### Etymology.

The name *Oenomaus myrteana* is intended to highlight the striking resemblance between the ventral hindwing of this species and that of *Enos myrtea* (Hewitson). The name is a feminine noun in apposition.

#### Habitat and distribution.

*Oenomaus myrteana* occurs in lowland wet forest from eastern Ecuador to western Brazil (Rondônia) (Fig. 46). Busby observed males in Ecuador low in the understory at 11:00 hours. This species and *Enos myrtea* have been found at the same site.

### Oenomaus mentirosa

Faynel & Robbins sp. n.

urn:lsid:zoobank.org:act:FDD83214-61B2-4B8A-B468-984D9DA0560D

http://species-id.net/wiki/Oenomaus_mentirosa

Figs 7 , 23 , 40 , 47

#### Type-locality.

Peru: Madre De Dios, Río La Torre, Tambopata Res., 12°50'13"S, 69°17'35"W, 300 m. Tambopata is at the mouth of the Río La Torre. In 1986 there was a lodge and a network of trails through uncut wet lowland forest. The holotype was collected during the transition between the dry and wet seasons when butterfly abundance and diversity generally peak.

#### Type-specimen.

**Holotype** ♂ (Fig. 7) labeled as “PERU Madre De Dios / Rio La Torre 300m / Tambopata Res. / 3 Oct.’ 86 / S. S. Nicolay” [rectangular, white, printed and handwritten], “GENITALIA NO. / 2011: 409♂ / C. FAYNEL” [rectangular, green, printed] “Holotype ♂ / *Oenomaus mentirosa* / Faynel & Robbins, 2012” [rectangular, red, printed]. Deposited in USNM.

**Paratypes: Peru.** **4** ♂: LO, km 28, Iquitos-Nauta, 180 m, 0359/7326, 30.X.2003, J.J. Ramírez leg. (MUSM) ; MD, Boca Río La Torre, 300 m, 17.IX.1984, I. Bohórquez leg., Genitalia NO. 1992: 47♂ R.K. Robbins (MUSM); MD, Boca Río La Torre, 300 m, 27.X.1981, G. Lamas et al., Genitalia NO. 1992: 48♂ R.K. Robbins (MUSM) ; MD, Tambopata Reserve, 12°50'S, 69°17'W, 300 m, 27.X.1990, Leg. R. Robbins, Genitalia NO. 1992: 39♂ R.K. Robbins (USNM ENT 00180049).

#### Description, diagnosis and recognition as a distinct species.

Male FW length: 14.9 mm (SD = 0.3, *N* = 2). Wing pattern (Fig. 7) and genitalia (Fig. 23) illustrated. The ventral wing pattern of *Oenomaus mentirosa* is very similar to some species of the *Porthecla gemma* group (Faynel et al. 2011), but the male genitalia have the non-triangular bifurcate valvae in lateral aspect that are characteristic of *Oenomaus*. Its genitalia, especially the valvae, are very similar to those of *Oenomaus cortica* (D’Abrera) and *Oenomaus druceus* Faynel & Moser. *Oenomaus mentirosa* is the only known *Oenomaus* species with red scales at the base on the VHW. In addition, it has a distinctive white spot along the VFW costa in cell Sc-R1. This feature occurs in no other Eumaeini except *Porthecla minyia* (Hewitson) where there are two white markings placed side by side in the cell between the costa and Sc. In male *Oenomaus* species, the eighth tergum is generally rectangular, but the anterior and posterior edges may be modified. In *Oenomaus mentirosa*, the male eighth tergum has a slightly modified anterior edge which looks like a shallow “W”. The female of *Oenomaus mentirosa* is unknown.

#### Etymology.

The name of this species comes from the Spanish word ‘mentirosa’, which means a feminine liar. We picked this name because the underside wing pattern resembles that of *Porthecla gemma* (Druce) and *Porthecla minyia* (Druce), but this resemblance appears to be a false indicator of relationship. We treat the name as a feminine noun in apposition.

#### Habitat and distribution.

*Oenomaus mentirosa* is known from lowland wet forest in Amazonian Peru (Fig. 47).

#### Remarks.

Resemblance of the ventral wing patterns of *Oenomaus mentirosa* and *Porthecla gemma*/*Porthecla minyia* was noted in the etymology. Adults of all three species fly in the same habitats at the same time of year in the vicinity of Puerto Maldonado, Peru.

### Oenomaus andi

Busby & Faynel sp. n.

urn:lsid:zoobank.org:act:C6C09BC9-F0B9-4E7A-8951-0D9923BE9E73

http://species-id.net/wiki/Oenomaus_andi

Figs 8, 9 , 24 , 29 , 42, 43 , 47

#### Type-locality.

Ecuador: Zamora Chinchipe Prov., Zamora (ridge W. of town), 4°04.5'S, 78°58.1'W, 1450 m. The ridge west of Zamora rises rather sharply from the city and is accessed by a dirt road which goes up to about 1300 m. The top of the ridge is still forested but a significant part of the surrounding land has been turned into pasture.

#### Type-specimen.

**Holotype** ♂ (Fig. 8) labeled as “ECUADOR / Zamora Chinchipe Prov. / Zamora (ridge W. of town) / 18. ix. 2000 (1450m) / leg. Robert C. Busby” [rectangular, white, printed], “GENITALIA NO. / 2009: 344♂ / C. FAYNEL” [rectangular, green, printed] “Holotype ♂ / *Oenomaus andi* / Busby & Faynel, 2012” [rectangular, red, printed]. Deposited in USNM.

**Paratypes: Ecuador.** **3**♀: Morona-Santiago, 1 km E Río Abanico, 1600 m, 2°15.4'S; 78°11.7'W, 15.IX.2003, Robert C. Busby leg., gen. prep. CF n°416 (RCB) (Fig. 9) ; Morona-Santiago, 14 km W. of Macas, 1600m, 28.IX.1998, Río Abanico, leg. Robert C. Busby (RCB); Zamora Chinchipe, Zamora (ridge W. of town), 4°04.5'S, 78°58.1'W, 1450 m, 06.X.2007, D. H. Ahrenholz, R. C. Busby leg. (RCB).

#### Other specimen examined.

**Bolivia**. **1**♀: La Paz, Nor Yungas, Caranavi, 1500 m, XII. 2004, gen. prep. CF n°445 (MC 253).

#### Description, diagnosis and recognition as a distinct species.

Male FW length: 16.3 mm (*N* = 1). Female FW length: 16.7 mm (SD = 0.8, *N* = 2). Wing pattern (Figs 8, 9) and genitalia (Figs 24, 29) illustrated. The ventral wing pattern of *Oenomaus andi* is similar to that of many other *Oenomaus*, but this species is distinguished by (1) a white spot on the basal side of VHW cell Rs-M1, (2) an elongated double valvae of equal size, (3) a large posterior part of the saccus in lateral view, (4) a swollen terminal end of the penis, and (5) modified anterior and posterior edges of the male 8th tergum (detailed under remarks).

#### Etymology.

This species is named for Andrea (Andi) Busby, wife of Robert Busby, in appreciation for her long standing support of his research. The name is a feminine noun in apposition.

#### Remarks.

Valvae structure in *Oenomaus andi* is very similar to that found in *Oenomaus gaia* Faynel, suggesting that this new species belongs to the *Oenomaus cortica* subgroup (as characterized by Faynel and Moser 2008). Species in this subgroup have a modified 8th tergum (except for *Oenomaus druceus* Faynel & Moser, 2008). In the male of *Oenomaus andi* (Fig. 42), the posterior edge of the 8th tergum has a deep depression in the middle, while the anterior edge is shaped like a wide “W”. In the female, the posterior edge is nearly straight but is split in the middle. The anterior edge is similar to that of the male, but is laterally sclerotized (Fig. 43). The white spot on the basal side of VHW cell Rs-M1 occurs in only a few other *Oenomaus* species including *Oenomaus geba* (Hewitson), *Oenomaus melleus* (Druce), *Oenomaus morroensis* Faynel & Moser, and *Oenomaus jauffreti* Faynel & Moser. Regardless of whether the presence of this spot is evidence of relationship, it is very useful for separating *Oenomaus andi* from the other species of the *Oenomaus cortica* subgroup.

#### Habitat and distribution.

*Oenomaus andi* is a species of montane forest (> 1300 m) that is recorded from Ecuador to Bolivia (Fig. 47).

#### Behavior.

A male and two females were attracted to traps baited with rotting fish (vouchers in RCB).

### Oenomaus moseri

Robbins & Faynel sp. n.

urn:lsid:zoobank.org:act:DC5D1BF6-2149-4BB9-802D-58C27D11AB38

http://species-id.net/wiki/Oenomaus_moseri

Figs 10, 11 , 25 , 30 , 44, 45 , 46

#### Type-locality.

Brazil: SC, Joinville, 26°19'39"S, 48°57'38"W, 10–200 m. Miers collected butterflies for decades in the wet lowland forests around Joinville, where he lived. His favorite collecting spot was a hill that he called “Serrinha” (little hill in Portuguese) in Vila Nova, approximately 10 km west, south-west of the center of Joinville. According to DZUP butterfly curator Olaf Mielke, specimens collected on Serrinha, including the holotype, have an elevation label 10–200 m, which distinguishes them from those specimens collected in other parts of the Joinville area.

#### Type-specimen.

**Holotype** ♂ (Fig. 10): **Brazil**, SC, Joinville, 10–200 m, 2.IV.1978, Miers leg.,gen. prep. CF n°218, DZ 10.065, CF-LYC-012 (DZUP).

**Paratypes: Brazil.** **12** ♂: SC, Joinville, 200 m, 26°19'S, 48°58'W, 20.V.1971, H.Miers leg., gen. prep. CF n°444 (MC 252) ; SC, São Bento do Sul, 600 m, 25.IV.2002, Moser & Rank leg., gen. A. Moser, n°234 (MC 034) ; SC, Joinville, 200 m, 5.II.1993, A. Moser leg.,gen. A. Moser, n°226 (MC 032); SC, Joinville, 200 m, 5.II.1993, A. Moser leg.,gen. A. Moser n°233 (MC 033) ; SC, Joinville, 10–200 m, 8.XII.1983, Leg. H. Miers, R.K. Robbins collection (USNM) ; SC, Joinville, 10–200 m, 6.I.1984, Leg. H. Miers, R.K. Robbins collection (USNM) ; PR, Ponta Grossa, Buraco do Padre, 900 m, 20.II.2009, Carlos Mielke leg., CF-LYC-063 (CF) ; SP, Serra do Japi, 110[0m], 23°15'S, 46°54'W, 12.IV.1991, Robbins & K. Brown, territorial behavior at 14:23, Genitalia NO. 1992: 27♂ R.K. Robbins (USNM) ; SP, Serra do Japi, 110[0]m, 23°15'S, 46°54'W, 12.IV.1991, Robbins & K. Brown, territorial behavior at 14:48 (USNM) ; SP, Serra do Japi, 800–1250 m, 23°12'S, 47°02'W, 23°17'S, 46°53'W, 25.III.1990, Leg. K. Brown (x2, USNM) ; SP, Serra do Japi, 800–1250 m, 23°12'S, 47°02'W, 23°17'S, 46°53'W, 28.III.1990, Leg. K. Brown (USNM) ; RJ, Petrópolis, 6.I.1980, Leg. C. Callaghan, R.K. Robbins collection, Genitalia NO. 1992: 79♂ R.K. Robbins (USNM ENT 00180045). **1**♀: SC, Joinville, 10–200 m, 9.III.1973, Leg. H. Miers, R.K. Robbins collection, gen. prep. CF n°410 (USNM) (Fig. 11).

#### Description, diagnosis and recognition as a distinct species.

Male FW length: 16.1 mm (SD = 0.9, *N* = 8). Female FW length: 15.7 mm (*N* = 1). Wing pattern (Figs 10, 11) and genitalia (Figs 25, 30) illustrated. The adult wing pattern of *Oenomaus moseri* is similar to that of the sympatric *Oenomaus morroensis* Faynel & Moser and to that of *Oenomaus cyanovenata* (D’Abrera); the species with which it was previously confused (Faynel 2008). *Oenomaus moseri* (Figs 25, 45) differs from *Oenomaus morroensis* (plate 11 in Faynel and Moser 2008) by its male genitalia having (1) a smaller dorsal part of the valvae attached to the top of the ventral part, not to the bottom, (2) a swollen posterior part of the male penis, and (3) a larger posterior part of the saccus in lateral view. *Oenomaus moseri* differs from O. *cyanovenata* by (1) a wider DFW black margin at the tornus, (2) a central depression on the posterior edge of the eighth tergum, and (3) a swollen posterior part of the male penis. *Oenomaus moseri* differs from the sympatric *Oenomaus geba* by lacking a white spot on the basal side of VHW cell Rs-M1 (Figs 10–12). The lack of geographical variation in the characters distinguishing *Oenomaus moseri* and *Oenomaus cyanovenata* argues against the hypothesis that the former is a geographical variant of the latter.

Preliminary data on divergence of “barcode” DNA sequence data is consistent with morphology. The divergence among three individuals of *Oenomaus moseri* (CF-LYC-012 & CF-LYC-063) is 0%, among four individuals of *Oenomaus cyanovenata* (CF-LYC-047, CF-LYC-048, & CF-LYC-049) is 0%. In contrast, the divergence between *Oenomaus moseri* and *Oenomaus cyanovenata* is more than 4% and between two *Oenomaus moseri* and a paratype of *Oenomaus morroensis* (CF-LYC-015) is more than 5%.

#### Etymology.

It is with great pleasure that we name this distinctive species for our good friend and collaborator Alfred Moser. Alfred lives in Rio Grande do Sul and has made prodigious contributions to the knowledge of Lepidoptera from southern Brazil, including co-authoring papers on the taxonomy of *Oenomaus* and *Porthecla* (Faynel and Moser 2008, Faynel et al. 2011).

#### Biology.

Robbins observed two males of *Oenomaus moseri* exhibiting territorial behavior on a hill top from 14:23 hours to 14:48 hours at Serra do Japi (SP, Brazil) on 12 April 1991 (vouchers in USNM). A male of *Oenomaus moseri* was reared by Hipólito Ferreira Paulino Neto in Itirapina, SP, Brazil on *Duguetia furfuracea* (A. St. Hil) Benth. and Hook. f. (Annonaceae), a plant of frequent occurrence in the cerrado. We identified the male from a digital image and from the locality where it was reared. However, it is possible that it is a male of *Oenomaus morroensis*, even though this species is not known to occur as far north as São Paulo.

#### Habitat and distribution.

*Oenomaus moseri* occurs in lowland and lower montane forest in southern Brazil (Fig. 46).

## New data for previously described speciesof *Oenomaus*

For each of the 21 previously described *Oenomaus* species, we give distribution, habitat, and remarks. We then note, where relevant, new information on taxonomy, intraspecific variation, behavior/biology, associated females, and COI DNA sequences. The species are treated in alphabetical order. *Oenomaus curiosa* and *Oenomaus melleus* are included in this section, even though their generic placement is yet unresolved (Faynel et al. 2011).

### Oenomaus ambiguus

Faynel

http://species-id.net/wiki/Oenomaus_ambiguus

#### Distribution, habitat, and remarks.

*Oenomaus ambiguus* is a poorly known, lowland species whose ventral wing pattern is virtually indistinguishable from those of *Oenomaus cortica* and *Oenomaus gaia*. It has been recorded from French Guiana and Amazonian Peru. The previous record from Amazonas, Brazil (Faynel 2008) was incorrect.

#### New material examined. French Guiana

. **1**♂: Bas Maroni, Guyane Française, gen. prep. CF n°319 (MNHN H-452). **Peru**.– **1**♂: MD, Río La Torre, 300 m, Tambopata Res., 27.IX.1987, S.S. Nicolay, gen. prep. CF n°404 (USNM).

#### Female.

Unknown.

#### COI DNA sequence.

The paratype from Peru has been barcoded (CF-LYC-025), and the sequence is 2–3% divergent from those of *Oenomaus cortica*, *Oenomaus gaia* and *Oenomaus morroensis* (Table 1).

### Oenomaus atena

(Hewitson)

http://species-id.net/wiki/Oenomaus_atena

#### Distribution, habitat, and remarks.

*Oenomaus atena* is a widely distributed lowland species that is reliably recorded from Costa Rica, Panama, western Ecuador, French Guiana, Venezuela, Peru, and Brazil (AM, MT). Most species with an “*atena*-like” ventral wing pattern have historically been identified as *Oenomaus atena*, which means that virtually all literature records for *Oenomaus atena* from before 2005 are unreliable.

#### New material examined.

**Costa Rica**.– **1**♂: Guápiles, 850 ft. alt., June, Schaus and Barnes coll., genitalia on slide X-10-1946, W.D.F. 2333 (USNM). **Panama**.– **1**♂: Cerro Campana, 2000’, XII-22-1963, G.B. Small, Genitalia 1992: 15♂ R.K. Robbins (USNM). **Ecuador**.– **1**♂: Esmeraldas, 25 km San Lorenzo-Lita Road, 1°10.0'N, 78°40.0'W, 100 m, VI.2003, San Francisco, R. Aldas & Robert C. Busby leg., gen. prep. CF n°343 (RCB). **Peru**.– **2**♂: MD, 30 km S.W. Pto. Maldonado, 300 m, 20.X.1983,S.S. Nicolay, Genitalia 1992: 16♂ R.K. Robbins (USNM); MD, 10 km north Puerto Maldonado, 200 m, 12°36'S, 69°11'W, 26–30.XI.1993, leg. C. Tello (USNM).

#### Female.

The female of this species was determined by a pair collected *in copula* and was illustrated by Faynel (2008, fig. 2).

#### COI DNA sequence.

Three specimens of *Oenomaus atena* have been barcoded, including a male from Peru (LO) (CF-LYC-084) and two females from French Guiana (CF-LYC-054 and CF-LYC-057). The latter two have the same brown dorsal wing pattern, ventral wing pattern, and genitalia as the female of *Oenomaus atena* found *in copula*. The three barcodes show 0.4% divergence.

### Oenomaus atesa

(Hewitson)

http://species-id.net/wiki/Oenomaus_atesa

#### Distribution, habitat, and remarks.

*Oenomaus atesa* is a widespread species that has been recorded from Mexico, Panama, western Ecuador, French Guiana, Venezuela, Colombia, eastern Ecuador, Peru, and Brazil (AM, DF, MG, RJ, SP, SC). The vast majority of museum specimens were collected in the lowlands, but males have also been found at 1375–1700 m in western Ecuador and at 2200 m in western Colombia (Prieto and Dahners 2006).

#### New material examined.

**Venezuela**.– **1**♀: Venezuela, Aragua, Rancho Grande, 1100 m, 29.V.1985, S.S. Nicolay leg., gen. prep. CF n°404 (USNM). **Ecuador**.– **2**♂: Pichincha 5 km Nanegal-García Moreno Rd, 0°09.2'N, 78°39.4'W, 4.VI.2008, 1375–1700 m, Robert C. Busby leg., gen. prep. CF n°340 (RCB);Napo Province, 14 km S of Tena, 17–18.X.1996, 600 m, Robert C. Busby leg., gen. prep. CF n°347 (RCB). **1**♀: Río Chuchuví, Lita vers San Lorenzo km12, 700 m (provincia de Esmeraldas), VIII.2001, Euclides Aldaz leg. (PB). **Peru**.– **1**♀: LO, 180 m, San Salvador, 5 km NNW Contamana, 08°19'S, 75°01'W, 27.XI.2002, D.H. Ahrenholz leg., gen. prep. CF n°403 (USNM). **Brazil**.– **1**♂: DF, Parque do Gama, 950 m, 14.V.1969, S.S. Nicolay leg., gen. prep. CF n°405 (USNM ENT 00180586).

#### Intraspecific variation.

Despite substantive geographical variation in *Oenomaus atesa*, we lack sufficient material to determine if this variation might represent more than one species. Females from Venezuela and western Ecuador have more extensive dorsal blue and a somewhat lighter color than females from Panama, French Guiana, eastern Ecuador, and Peru. In addition, males from western Ecuador have more blue on the dorsal forewings than males from eastern Ecuador. However, this variation is small compared to that between males of *Oenomaus atesa* and *Oenomaus lea*. For example, the forewing dorsal blue area never reaches the cells from vein R3 to Cu1 as it does in *Oenomaus lea*. Structure of the female genitalia also varies geographically. Females from Venezuela and Peru have two processes at the posterior end of the lamella postvaginalis while a female from French Guiana had none (see Faynel 2006, p. 29).

#### Behavior/biology.

Males exhibited territorial behavior on a hilltop in Panama (Canal Area, Gamboa, Cerro Pelado) from 13:15 to 15:30 hours (19 males, 10 different days during the months of January, February, March, April, August, September, October, and December, 15 vouchers in USNM). Similarly, territorial males on a hilltop in Brazil (Santa Catarina, Villa Nova, Serrinha) were observed from 14:40 to 14:55 hours (3 males, March, 3 vouchers in USNM).

#### Female.

Females are associated with males by their ventral wing pattern, which is unique among the Eumaeini. Characters were noted for distinguishing the ventral wing pattern of *Oenomaus atesa* from that of *Oenomaus lea*.

#### COI DNA sequence.

One male of *Oenomaus atesa* from French Guiana has been barcoded (CF-LYC-003).

### Oenomaus brulei

Faynel

http://species-id.net/wiki/Oenomaus_brulei

#### Distribution, habitat, and remarks.

Faynel (2008) described *Oenomaus brulei* from one male collected in the lowlands of French Guiana. Since then, another male and female from French Guiana have been examined.

#### New material examined.

**French Guiana**.– **1**♂: Guyane, no date, S. Fernandez leg., CF-LYC-033 (CF). **1**♀: Montagne des Singes, 5°07'N, 52°69'W, 5.XII.2007, T. Rosant leg., gen. prep. CF n°440, CF-LYC-034 (CF) (Fig. 14).

**Female**. We associate a female (Figs 14, 31) which has the same ventral wing pattern as the male, which occurs in French Guiana (as do the known males), and which has a very similar COI DNA sequence to that of the males.

#### COI DNA sequence.

Divergence among the three known specimens is 0.2%.

### Oenomaus cortica

(D’Abrera)

http://species-id.net/wiki/Oenomaus_cortica

#### Distribution, habitat, and remarks.

This species occurs in wet lowland forest and is recorded from Panama, Guyana, Peru, and Brazil (PA, AM). *Oenomaus cortica*, *Oenomaus gaia*, and *Oenomaus ambiguus* have very similar wing patterns, but their genitalic structures are distinct.

#### New material examined.

**Panama**.– **1**♂: Gatún, C. Z., 2.V.1970, G.B. Small leg., Genitalia 1992: 13♂ R.K. Robbins (USNM). **Guyana**.– **1**♂: Potaro Riv., VIII-IX.1902, C.B. Roberts, Genitalia 1992: 74♂ R.K. Robbins (FSMC). **Peru**.– **1**♂: MD, Parque Manu, Pakitza 340 m, 11°55'48"S, 71°15'18"W, 14.X.1991, Leg. R. Robbins, Genitalia No. 1996: 3♂ R.K. Robbins (USNM ENT 00180044).

#### Intraspecific variation.

The male from Panama has the posterior edge of its 8th tergum more deeply incised than in others.

#### Female.

Unknown. A female paratype of *Oenomaus cortica* from Espírito Santo, Brazil was illustrated in D’Abrera (1995), but no definitive evidence was presented to support this identification.

#### COI DNA sequence.

Two males from Brazil, Pará have been sequenced (CF-LYC-051 and CF-LYC-052) and show 0.6% divergence.

### Oenomaus curiosa

Faynel & Moser

http://species-id.net/wiki/Oenomaus_curiosa

#### Distribution, habitat, and remarks.

*Oenomaus curiosa* is a species of wet lowland forest that is recorded from French Guiana, Peru (LO, MD), and Brazil (RO).

#### New material examined.

**Peru**.– **2**♂: MD, 300 m, 30 km S. W. Pto Maldonado, 26.X.1983, S.S. Nicolay, Genitalia No. 1992: 25♂ R.K. Robbins (USNM); LO, 120 m, Pebas, river Amazonas, 03°19'S, 71°51'W, II. 2011, Ramírez leg. (CF). **Brazil**.– **1**♂: RO, 62 km SW Ariquemes, Línea 20, lot 21, 23, 25 (Fazenda Rancho Grande), 11.X.1993, AVZ Brower, gen. prep. CF n°433 (OSAC).

#### Female.

Unknown

#### COI DNA sequence.

Two males from French Guiana, including one of the paratypes, have been sequenced (CF-LYC-036 and CF-LYC-037) and show 0.8% divergence.

### Oenomaus cyanovenata

(D’Abrera)

http://species-id.net/wiki/Oenomaus_cyanovenata

#### Distribution, habitat, and remarks.

A species of very wet lowland forest, it has been recorded from Costa Rica, Panama, French Guiana, Venezuela, Bolivia, and Brazil (PA, AM). The previous record for Brazil (SC) was incorrect; this specimen is now treated as *Oenomaus moseri*.

#### New material examined.

**Costa Rica**.– **1**♂: Guápiles, 850 ft. alt., Schaus and Barnes coll., Genitalia 1992: 76♂ R.K. Robbins (USNM); **2**♀: Area de Conservación Guanacaste, voucher: D.H. Janzen & W. Hallwachs 97-SRNP-62841.1, Genitalia 2009: 30♀ R.K. Robbins (USNM) (Fig. 16); 97-SRNP-6283. **Panama**.– **1**♂: Colón, Piña, 100 m, 9.IV.1971, H.L. King, genitalia slide/vial #4710, prep. S.S. Nicolay (USNM). **French Guiana**.– **3**♀: Roura, Route de Kaw - PK 16, 18.VII.2004, C. Faynel leg., CF-LYC-053 (CF); Roura, Route de Kaw, 26.I.2005, J.Y. Gallard leg., gen. prep. CF n°441, CF-LYC-055 (CF) (Fig. 17); Roura, Route de Kaw - PK 8, 20.XII.2001, J.Y. Gallard leg., CF-LYC-056 (CF). **Brazil**.– **3**♀ : PA, Santo Antônio do Tauá, Reserva Sonho Azul, 1°15'S, 48°03'W, 12.VI.2009,P. & J. Jauffret leg., CF-LYC-059 (CF); PA, Santo Antônio do Tauá, Reserva Sonho Azul, 1°15'S, 48°03'W, 3.VIII.2009,P. & J. Jauffret leg., CF-LYC-060 (CF); PA, Santo Antônio do Tauá, Reserva Sonho Azul, 1°15'S, 48°03'W, 8.V.2009,P. & J. Jauffret leg., CF-LYC-061 (CF).

#### Intraspecific variation.

Females from French Guiana and Brazil, Pará (Fig. 17) are uniformly brown on the dorsal wing surface while the female from Costa Rica (Fig. 16) has the basal parts of both wings blue. Their genitalia, however, are uniform. Additionally, their COI DNA sequences are similar. This geographic variability is similar to that in *Oenomaus taua*.

#### Behavior/biology.

Two females were reared in Costa Rica (97-SRNP-62841.1 and 97-SRNP-6283) from *Guatteria verrucosa* R.E. Fr. (Annonaceae) (adult vouchers in USNM). Details of the rearing records along with images of the immatures can be found in Janzen and Hallwachs (2012).

#### Female.

Females of this species (Figs 16, 17, 32, 33) have the same ventral wing pattern as males, occur at the same localities, and have similar COI DNA sequences. A female paratype of *Oenomaus cyanovenata* from Pará, Brazil was designated and illustrated in D’Abrera (1995) without definitive supporting evidence. This female has a different dorsal wing pattern than the female from Pará that we have associated with the male. We are skeptical of the biological validity of this paratype designation.

#### COI DNA sequence.

Four males and seven females from French Guiana and Brazil, Pará were barcoded. One male (CF-LYC-046) is 6.7% divergent from the other three males, but its sequence is identical with that from a male of *Oenomaus magnus* (CF-LYC-020). Potential explanations for this result range from contamination to biologically significant, but until we have additional information, we omit this male from the following results. Divergence among the 10 other specimens of *Oenomaus cyanovenata* was 0.1%. The reared females from Costa Rica, which were barcoded in another project, are 0.4% divergent from the South American specimens.

### Oenomaus druceus

Faynel & Moser

http://species-id.net/wiki/Oenomaus_druceus

#### Distribution, habitat, and remarks.

This species was described from one Brazilian (AM) male, which is the only known specimen. As noted, its genitalia are similar to those of *Oenomaus mentirosa*, but it has a distinctly different ventral wing pattern.

#### Female.

Unknown.

### Oenomaus floreus

(Druce)

http://species-id.net/wiki/Oenomaus_floreus

#### Distribution, habitat, and remarks.

This species occurs in lowland and lower montane habitats with wet or deciduous forest. It is recorded from eastern Ecuador and Brazil (AM, MT, DF, GO, PR).

#### New material examined.

**Ecuador**.– **1**♂: Pastaza Province, 45 km Puyo-Arajuno Rd., 1000 m, 26.IX.1999, Robert C. Busby leg., gen. prep. CF n°342 (RCB). **Brazil**.– **2**♂: GO, 163 km W. Jataí S. Rita Araguaia, 850 m, 29.V.1969, S.S. Nicolay, genitalia slide/vial #4367, prep. S.S. Nicolay (USNM); PR, Highlands, 24.XI.1934, coll. Karl Schmitt, E.I. Huntington, Genitalia 1992: 19♂ R.K. Robbins (AMNH).

#### Female.

Described by Faynel and Moser (2008).

### Oenomaus gaia

Faynel

http://species-id.net/wiki/Oenomaus_gaia

#### Distribution, habitat, and remarks.

This species occurs in wet and dry lowland forest. It has been recorded from Panama, French Guiana, Venezuela, eastern Ecuador, Peru (LO, SM, UC, MD) and Brazil (PA, AM, RO, MT, GO). This species, *Oenomaus floreus*, and maybe *Oenomaus griseus* occur in drier forest than other species with an “*atena*-like” ventral wing pattern.

#### New material examined. 

**Panama**.– **1**♂: Los Ríos, C. Z., 15.XII.1964, S.S. Nicolay leg., Genitalia 1992: 73♂ R.K. Robbins (USNM ENT 00180046). **2**♀: Los Ríos, C. Z., 27.I.1965, S.S. Nicolay, gen. prep. CF n°430 (USNM) (Fig. 15); Los Ríos, C. Z., 19.XII.1964, G.B. Small, gen. prep. CF n°431 (USNM). **Ecuador**.– **1**♂: Morona-Santiago 15 km S Gualaquiza, 850 m, 3°27.6'S, 78°33.1'W, 27.IX.2000, Robert C. Busby leg. (RCB). **Peru**.– **1**♂: MD, Parque Manu, Pakitza 340 m, 11°55'48"S, 71°15'18"W, 15.X.1991, Leg. M. Casagrande, Genitalia No. 1992: 38♂ R.K. Robbins (USNM). **Brazil**.– **2**♂: PA, Obidos, IX.1930, Ex coll. Le Moult, Genitalia No. 1992: 75♂ R.K. Robbins; PA, Santo Antônio do Tauá, Reserva Sonho Azul, 1°15'S, 48°03'W, 16.VII.2003,P. & J. Jauffret leg., CF-LYC-072 (CF); RO, 62 km SW Ariquemes, Línea 20, lot 21, 23, 25 (Fazenda Rancho Grande), 11.X.1993, AVZ Brower, gen. prep. CF n°411 (OSAC); GO, Pirenópolis, 820 m, 15°49'S, 48°59'W, E. Emery leg. (MC 255).

#### Female.

Four males in the USNM were collected on hills on Los Ríos hill (approximately 9°00'32"N, 79°35'34"W) and in Cocolí (approximately 8°58'46"N, 79°35'59"W), Canal Area, Panama. These areas are drier (<2 m annual precipitation, Rand and Rand 1982) than the forest in which other *Oenomaus* with an “*atena*-like” wing pattern have been found in Panama. Four females from these two localities have the same ventral wing pattern as the males. Since no other males are known from these localities, we associate the sexes and illustrate the adult wing pattern and genitalia of one of these females (Figs 15, 34).

We also associate a female from Brazil, Pará (CF-LYC-072) with a male of *Oenomaus gaia* from French Guiana because they have the same ventral wing pattern and have similar barcode sequences (0.2%).

#### COI DNA sequence.

As noted previously, interspecific variation in the barcode sequences of *Oenomaus ambiguus*, *Oenomaus cortica*, *Oenomaus gaia*, *Oenomaus morroensis* is less than 2%, in contrast to interspecific divergences among other species in *Oenomaus*. For example males of *Oenomaus gaia* (CF-LYC-023) and *Oenomaus cortica* (CF-LYC-052) are 0.8% divergent. Another male of *Oenomaus gaia* (CF-LYC-024) and *Oenomaus morroensis* (CF-LYC-015) are 1.1% divergent.

### Oenomaus geba

(Hewitson)

http://species-id.net/wiki/Oenomaus_geba

#### Distribution, habitat, and remarks.

This species is a relatively uncommon inhabitant of lower montane forest in southern Brazil, so far known only from the state of Santa Catarina. Previously, it was known only from the male holotype, which lacks locality data.

#### New material examined.

**Brazil**.– **5**♂: SC, Highlands near Massaranduba-Blumenau, Collection E.I. Huntington No. 1009 (AMNH, x4); SC, Highlands near Massaranduba-Blumenau, Collection E.I. Huntington No. 1009, genitalia slide/vial #4705, prep. S.S. Nicolay, Allyn Museum Photo No. 022078-7, 8 VI (AMNH) . **2**♀: SC, Rio Vermelho, São Bento do Sul, 10.III.1973, leg. Rank, gen. prep. CF n°414 (USNM ENT 00180041); SC, Highlands near Massaranduba-Blumenau, Collection E.I. Huntington No. 1009, genitalia slide/vial #4707, prep. S.S. Nicolay, Allyn Museum Photo No. 022078-9, 10 VI (AMNH).

#### Female.

The female (Figs 12, 35) occurs in the same habitat as the male and has the same ventral wing pattern.

### Oenomaus griseus

Faynel & Moser

http://species-id.net/wiki/Oenomaus_griseus

#### Distribution, habitat, and remarks.

This species appears to be endemic to Brazil’s central plateau (DF).

#### Female.

Unknown.

### Oenomaus isabellae

Faynel

http://species-id.net/wiki/Oenomaus_isabellae

#### Distribution, habitat, and remarks.

This widespread South American species occurs in wet and dry lowland forests. It is recorded from French Guiana, Colombia, eastern Ecuador, Peru, Bolivia, and Brazil (AM, MG).

#### New material examined.

**Colombia**.– **1**♀: Muzo, 400b. 800 m, coll. Fassl (SMF). **Ecuador**.– **2**♀: Morona-Santiago, Santiago (Hill North of Town), 3°02.3'S, 78°00.3'W, 350 m, 20.IX.2010, Robert C. Busby leg. (RCB); 27 km Santiago-Puerto Moreno Rd., 2°56.4'S, 77°49.5'W, 500–550 m, 17 IX 2005, Robert C. Busby, leg. (RCB). **Peru**.– **1**♀: JU, Aldea, 600–700 m, 1054/7455, 23.VIII.2003, J.J. Ramírez (MUSM). **Brazil**. – **2**♂: MG, km 500 Belo Horizonte-Brasília, Hwy, 11.IV.1973, C. Callaghan, genitalia slide/vial #4737, prep. S.S. Nicolay (USNM); AM, Rio Amazonas, Vila Nova (ca. Tonantins, 0252S/6748), 100 m, IX.1993, M. Büche leg. (MUSM). **Bolivia**.– **1**♂: Las Juntas, XII. 1913, Steinbach Acc. 5045, genitalia slide/vial #4743, prep. S.S. Nicolay (CMNH).

#### Female.

The distinctive ventral wing pattern of this species allows identification of the female.

#### COI DNA sequence.

Sequences from a Brazilian male (CF-LYC-006) and French Guiana female paratype (CF-LYC-007) diverge 3.0%.

### Oenomaus jauffreti

Faynel & Moser

http://species-id.net/wiki/Oenomaus_jauffreti

#### Distribution, habitat, and remarks.

This species inhabits wet lowland forest. It is recorded from French Guiana, eastern Ecuador, Peru, Bolivia, and Brazil (PA, MT).

#### New material examined.

**Ecuador**.– **1**♂: Pastaza Province, 32 km S. of Puyo, 20–21.X.1996, 1000 m, Robert C. Busby leg., gen. prep. CF n°346 (RCB). **Peru**.– **1**♂: LO, Agua Blanca, 0356/7328, 130 m, 10.XI.2005, J.J. Ramírez (MUSM). **2**♀: JU, vic. Satipo, c. 800 m, Villa Esperanza, c. 11°16'S, 74°15'W, V.1983, leg. M. Callegari (USNM) ; LO, Cerros de Contamana, El Indio, 200 m, 10.IX.1986, P. Hocking (MUSM). **Bolivia**.– **1**♂: Río Songo, 750 m, coll. Fassl, Genitalia No. 2002: 5♂ R.K. Robbins (SMF).

#### Intraspecific variation.

As noted by Faynel and Moser (2008), *Oenomaus jauffreti* is a variable species, especially ventrally. For example, the VHW basal spot in cell Sc+R1 is large and mostly white in French Guiana; is small, black with a white centered pupil in Brazil (MT), and is large, with black and white scales in Ecuador. The only element which seems to be stable is the presence of a white spot on the basal side of VHW cell Rs-M1.

#### Female.

Females were associated with males by their characteristic ventral wing pattern (Faynel and Moser 2008). Six specimens of *Oenomaus jauffreti* have been barcoded (four males and two females), including three male paratypes (CF-LYC-028, CF-LYC-029, CF-LYC-030) and one female paratype (CF-LYC-032). The six barcodes show 1.2% divergence.

### Oenomaus magnus

Faynel & Moser

http://species-id.net/wiki/Oenomaus_magnus

#### Distribution, habitat, and remarks.

This is a poorly understood species that occurs in South American lowland forest. It has been recorded from French Guiana, Peru, Bolivia, and Brazil (AM, MT).

#### New material examined.

**French Guiana**.– **1**♀: Approuague - Mapaou, 4°31'N, 52°13'W, 29. XII. 2008, S. Fernandez leg. (CF) (Fig. 13). **Peru**.– **1**♀: SM, Upper Huallaga Valley, V-VI 2000, Purch. Thorne 7/01, gen. prep. CF n°428 (USNM). **Brazil**.– **1**♂: MT, Diamantino, Alto Rio Arinos, 5.X.1998, E. Furtado leg., gen. prep. CF n°446 (MC 254). **Bolivia**.– **1**♀: “Thecla melleus Drc.♀, Buenavista 750 m, Bolivia Steinbach., Modt. 22/2 1927 af, qui Steinbach Bolivia, Coll. C.S. Larsen, Faaborg, gen. prep. CF n° 449 (MNHN).

#### Female.

We associate females from French Guiana (Figs 13, 36), Peru, and Bolivia with this species. They have the same ventral wing pattern, a similar geographic range, and limited COI DNA sequences are the same.

#### COI DNA sequence.

The sequence of a female from French Guiana (CF-LYC-039) is the same as (0% divergence) that of the male paratype from Peru (CF-LYC-020). This female is the first record of *Oenomaus magnus* from French Guiana.

### Oenomaus melleus

(Druce)

http://species-id.net/wiki/Oenomaus_melleus

#### Distribution, habitat, and remarks.

This species occurs in wet lowland forest. It is recorded from Nicaragua, Costa Rica, French Guiana, Guyana, Venezuela, Colombia, Peru, Bolivia, and Brazil (PA, PE, ES).

#### New material examined.

**Costa Rica**.– **2**♂: Heredia, prov. Finca La Selva, 3 km S. Puerto Viejo, 10°26'N, 84°01'W, 26.VII.1992, leg. H.A. Hespenheide (USNM) ; prov. Heredia, F. La Selva, 3 km S. Pto. Viejo, 10°26'N, 84°01'W, 5.IV.1985, H.A. Hespenheide (USNM). **1**♀: Area de Conservación Guanacaste, voucher Janzen & Hallwachs #97-SRNP-62841, legs away for DNA, Genitalia 2009: 30♀ R.K. Robbins (USNM) (Fig. 20). **Guyana**.– **1**♂: Region 7 Lower Cuyuni River nr. Arimu R. 100’, 6°34'N, 58°58'W, 2.IX.1991, leg. S. Fratello (USNM ENT 00180024). **Brazil**.– **1**♂: ES, Itaguassú, IX.1971, Paulo César Elias, A.C. Allyn Acc. 1971-38, genitalia slide/vial #4700, prep. S.S. Nicolay (USNM). **1**♀: PE, Recife, 5.I.1962, leg. Ebert (USNM).

#### Taxonomy.

Faynel (2007, 2008) partitioned this species into a Transandean Region (terminology from Brown 1982) nominate subspecies and an Amazonian Region subspecies *Oenomaus melleus guyanensis* based on size and color of scales at the base of the VFW. The male genitalia of each taxon were the same. As noted in the next paragraph, the new material examined does not confirm this recognition of two taxa. For example, the Costa Rican specimens resemble the Amazonian ones. For this reason, we synonymize *Oenomaus melleus guyanensis* Faynel with *Oenomaus melleus melleus* (Druce), **new synonym**.

#### Intraspecific variation.

The wing pattern of *Oenomaus melleus* is highly variable. The type from Colombia and two specimens from Nicaragua and Venezuela are relatively large (male FW length = 16.8 mm, SD = 1.3, *N* = 3). They have a white spot on the basal part of VHW cell Rs-M1, no reddish scales on the basal part of ventral wing, and a black spot in VHW cell Cu1-Cu2. The specimens from French Guiana, Guyana, Brazil (PA), Venezuela and Peru (UC) are smaller (male FW length = 14.1 mm, SD = 0.4, *N* = 5). They have a white spot on the basal part of VHW cell Rs-M1, reddish scales on the basal part of ventral wing, and no black spot in VHW cell Cu1-Cu2. The males from Costa Rica are also relatively small (male FW length = 14.7 mm, SD = 1.6, *N* = 3). They have no white spot on the basal part of VHW cell Rs-M1, no reddish scales on the basal part of ventral wing and a black spot in VHW cell Cu1-Cu2.

#### Female.

Described by Faynel (2008).

### Oenomaus morroensis

Faynel & Moser

http://species-id.net/wiki/Oenomaus_morroensis

#### Distribution, habitat, and remarks.

Described by Faynel and Moser (2008) from five males from Brazil (SC, RS), but no other specimens are known. It appears to be a species of lower montane and subtropical forest.

#### Intraspecific variation.

A small white spot on VHW cell Sc+R1-Rs that is displaced basally (Faynel and Moser 2008) is present in the holotype, but not in the paratypes.

#### Behavior/biology.

Although *Oenomaus morroensis* is unrecorded north of Santa Catarina, a reared male from São Paulo (see under *Oenomaus moseri*) could possibly be this species.

#### Female.

Unknown.

#### COI DNA sequence.

One paratype has been barcoded (CF-LYC-015). As already noted, this sequence is 5.0% divergent from the sympatric and superficially similar *Oenomaus moseri*.

### Oenomaus nigra

Faynel & Moser

http://species-id.net/wiki/Oenomaus_nigra

#### Distribution, habitat, and remarks.

This species occurs in wet lowland forest. It has been recorded from Peru and Brazil (AM). As noted, the genitalia of this species are similar to those of the newly described *Oenomaus myrteana*.

#### New material examined.

**Peru**.– **3**♂: LO, Agua Blanca, 0356/7328, 130 m, 17.V.2004, J.J. Ramírez leg. (MUSM, x3). **Brazil**.– **1**♂: AM, S. Paulo de Olivença, X.1983, Via Kesselring, Genitalia No. 1983: 133♂ R.K. Robbins (USNM ENT 00180054).

#### Female.

Unknown.

#### COI DNA sequence.

One male of *Oenomaus nigra* from Peru has been barcoded (CF-LYC-148).

### Oenomaus ortygnus

(Cramer)

http://species-id.net/wiki/Oenomaus_ortygnus

#### Distribution, habitat, and remarks.

This species occurs in many different habitats from sea level up to 1000 m. It is unique in the genus in that it is often found in highly disturbed habitats. It is the most common *Oenomaus* species in collections and has been recorded from the United States, Mexico, Guatemala, Honduras, Nicaragua, Costa Rica, Panama, French Guiana, Surinam, Guyana, Trinidad, Venezuela, Colombia, Ecuador, Peru, and many states throughout Brazil. As noted in the introduction, this species is a well-known pest of commercial Annonaceae.

#### Intraspecific variation.

The blacks spots on ventral wings vary in size and the blue on the dorsal wings vary from light cyan to dark purple. The “*Thecla lauta* Draudt” phenotype from western Mexico is smaller and duller than individuals from the remainder of its range.

#### Behavior/biology.

Males were territorial on hilltops between 14:00–15:15 hours in Panama (Canal Area, hilltops in Paraíso, 7 males during June and August, 6 vouchers in USNM) and between 14:29 and 15:15 on Serrinha in Brazil (hilltop in Santa Catarina, Villa Nova, 200 m, 3 males in March, vouchers in USNM).

#### Female.

Both sexes are recognized by their ventral wing pattern, which is unique in the genus.

#### COI DNA sequence.

Sequences from a Peruvian male (CF-LYC-147) and a Mexican male (CF-LYC-146) diverge 1.5%.

### Oenomaus poirieri

Faynel

http://species-id.net/wiki/Oenomaus_poirieri

#### Distribution, habitat, and remarks.

This species occurs in wet lowland forest. It has been recorded from French Guiana and Brazil (PA, AM).

#### New material examined.

**Brazil**.– **1**♂. Santarém, Amazons, A.H. Fassl, 3.IV.1920, gen. prep. CF n°317 (MNHN H-447).

#### Female.

Described by Faynel (2008).

### Oenomaus taua

Faynel & Moser

http://species-id.net/wiki/Oenomaus_taua

#### Distribution, habitat, and remarks.

This species is widespread in wet lowland forest. It is recorded from Guatemala, Panama, French Guiana, eastern Ecuador, Peru, and Brazil (PA, AM, RO). It is one of the more common species in the genus and mating pairs have been collected in Panama, Ecuador, and Brazil.

#### New material examined. 

**Guatemala**.– **1**♂: Cayuga, Sept., Schaus & Barnes coll., Genitalia No. 1992: 28♂ R.K. Robbins (USNM). **Panama**.– **2**♂: Canal Zone, Gamboa, 5.I.1979, Leg. R. Robbins, *in copula* 15:00, Genitalia No. 1982: 125♂ R.K. Robbins (USNM ENT 00180050); Canal Zone, Summit, 17.III.1979, Leg. R. Robbins, *in copula* 15:00, gen. prep. CF n°423 (USNM). **2**♀: Canal Zone, Gamboa, 5.I.1979, Leg. R. Robbins, *in copula* 15:00, Genitalia No. 1982: 126♀ R.K. Robbins (USNM ENT 00180051) (Fig. 18); Canal Zone, Summit, 17.III.1979, Leg. R. Robbins, *in copula* 15:00, gen. prep. CF n°424 (USNM). **Ecuador**.– **2**♂: Napo, 14 km Tena-Puyo Road, 1°06.7'S, 77°46.9'W, 600 m, X.2010 (Apuya), I. Aldas & Robert C. Busby leg., gen. prep. CF n°418 (RCB);Napo Province, 14 km S. of Tena, 600 m, 17–18.X.1996 (Apuya), mating pair, Robert C. Busby leg., gen. prep. CF n°345 (RCB). **1**♀: Napo Province, 14 km S. of Tena, 600 m, 17–18.X.1996 (Apuya), mating pair, Robert C. Busby leg., gen. prep. CF n°417 (RCB) (Fig. 19). **Brazil**.– **1**♂: RO, 160–350 m, vic. Cacaulândia, 10°32'S, 62°48'W, 19.X.1991,* in copula*, Leg. J. MacDonald, gen. prep. CF n°412 (USNM). **1**♀: RO, 160–350 m, vic. Cacaulândia, 10°32'S, 62°48'W, 19.X.1991, *in copula*, Leg. J. MacDonald, gen. prep. CF n°413 (USNM); PA, Santo Antônio do Tauá, Reserva Sonho Azul, 1°15'S, 48°03'W, 2.III.2010,P. & J. Jauffret leg. (CF).

#### Intraspecific variation.

Females from Brazil and Ecuador (Fig. 19) are uniformly brown on the dorsal wing surface while the female from Panama (Fig. 18) has the basal parts of both wings blue. Their genitalia, however, are uniform. This geographic variability is similar to that in *Oenomaus cyanovenata*.

#### Behavior/ biology.

Territorial behavior on a hilltop in Panama (Canal Area, Gamboa, Cerro Pelado) was observed in January and August at 15:00 hours (vouchers in USNM). Two mating pairs were also collected on the same hilltop in January and March at 15:00 hours (vouchers in USNM).

#### Female.

We illustrate adult females that were collected *in copula* (Figs 18–19) and the genitalia of one (Fig. 37).

#### COI DNA sequence.

A female from Brazil, Pará (CF-LYC-064), which has a wing pattern similar to the females collected *in copula*, is 3.1% divergent from a male from Peru (CF-LYC-085).

## Discussion

**Taxonomy**. A decade ago *Oenomaus* was a monotypic genus, but it now consists of 28 described species (albeit, it is still unclear if *Oenomaus melleus* and *Oenomaus curiosa* belong to *Oenomaus* or *Porthecla*). Further, if a phylogenetic analysis shows that *Porthecla* is paraphyletic in terms of *Oenomaus*, which is possible because *Porthecla* was distinguished by character states that may be plesiomorphic, then Oenomaus will be one of the most species-rich eumaeine genera with 40 species (Robbins 2004).

There are three biological reasons why the diversity of *Oenomaus* was not recognized until recently. First, about ¾ of the species have an indistinguishable, or barely distinguishable, ventral wing pattern that is similar to that of *Oenomaus atena* (e.g., Figs 12–19). Among species with this wing pattern, there is a great diversity of male genitalic forms that were first documented by Faynel (2006, 2008) and Faynel and Moser (2008). Second, the ventral wing pattern of a few species is different from that of *Oenomaus atena* (e.g., Figs 1–7), but similar to that of sympatric species that are now considered to be distantly related. For example, Draudt (1919–1920) in Seitz grouped *Oenomaus ortygnus* (the type species of *Oenomaus*), now placed in the *Panthiades* Section, with *Atlides rustan* (Stoll), now placed in the *Atlides* Section (Robbins 2004). Similarly, he placed *Oenomaus atesa* in a group with *Enos mazurka* (Hewitson) in the *Brangas* Section. *Oenomaus myrteana*, which is described in this paper, closely resembles *Enos myrtea* while *Oenomaus mentirosa*, also newly described, has a ventral wing pattern that resembles species in *Porthecla*, *Olynthus* Hübner,* Janthecla* Robbins & Venables, and *Atlides* Hübner (documented in Faynel et al. 2011). Third, many *Oenomaus* species are exceedingly rare in collections. Indeed, three species are still known from only one individual each.

**DNA barcoding**. Thirty-eight *Oenomaus* males belonging to 19 species have been successfully “barcoded” (>200 bp) (extraction and sequencing methods given in Hajibabaei et al. 2006). For those nine species for which there is more than one barcode (Table 1), intraspecific divergence calculated on the Bold web site (http://www.boldsystems.org/views/login.php) using the Kimura 2 parameter with sequences aligned by BOLD varied from 0% to 1.6%. Interspecific divergence (Table 1) varied from 0.8% to 9.7% (672 comparisons, mean distance: 6.1%). It was usually greater than 4% except in the *Oenomaus cortica* species group (*Oenomaus gaia*, *Oenomaus cortica*, *Oenomaus morroensis*), where it was about 1%. Similarity in COI sequences among closely related species is well-established (e.g., Burns et al. 2007).

**Male-female associations**. Associating males and females in *Oenomaus* is sometimes very difficult. Only eight of the 28 recognized species previously had the sexes associated. In this paper we associate the sexes of another ten species based on mating pairs collected *in copula* and on similarity of ventral wing patterns, habitats, geographic distributions, and mitochondrial COI DNA sequences. The DNA “barcodes” have great potential (e.g., Janzen et al 2009), especially if there are large samples from geographically diverse sites.

**Biology**. *Oenomaus ortygnus* is a well-known pest of cultivated soursop (also called guanábana, *Annona muricata* L., Annonaceae) and relatives (e.g., Dampf 1929, Fennah 1937, Ballou 1945, Guagliumi 1965, 1967, Araque 1967, d’Araújo e Silva et al. 1967–1968, Leal 1970, Kendall 1975, Domínguez 1978, Peña et al. 2002, Castañeda-Vildózola et al. 2011). As noted in the results of this paper, two other *Oenomaus* species have now been reared, and Annonaceae (*Duguetia*, *Guatteria*) is a food plant for each. Although data are yet too scanty to ask why *Oenomaus ortygnus* is the only *Oenomaus* species that has been recorded as a pest on cultivated Annonaceae, we note that it is also the only *Oenomaus* species that is regularly found in disturbed habitats.

Most *Oenomaus* species inhabit relatively undisturbed lowland wet forest, but some species seem to be restricted to other habitats. *Oenomaus andi* is montane, being found so far only above 1450 m, while *Oenomaus geba* is known only from lower montane forest. *Oenomaus morroensis* occurs so far only in subtropical and lower montane forest. *Oenomaus druceus* has been found only in scrubby deciduous forest. A number of other *Oenomaus* species have broader habitat requirements. Some wet lowland species also occur in montane habitats, for which *Oenomaus atesa* and *Oenomaus moseri* are representative examples. Others, such as *Oenomaus floreus*, *O. gaia*, and *Oenomaus isabellae*, may inhabit dry deciduous forest. As previously noted, *Oenomaus ortygnus* is the only *Oenomaus* species that is regularly found in both undisturbed and disturbed habitats.

## Supplementary Material

XML Treatment for Oenomaus mancha

XML Treatment for Oenomaus gwenish

XML Treatment for Oenomaus lea

XML Treatment for Oenomaus myrteana

XML Treatment for Oenomaus mentirosa

XML Treatment for Oenomaus andi

XML Treatment for Oenomaus moseri

XML Treatment for Oenomaus ambiguus

XML Treatment for Oenomaus atena

XML Treatment for Oenomaus atesa

XML Treatment for Oenomaus brulei

XML Treatment for Oenomaus cortica

XML Treatment for Oenomaus curiosa

XML Treatment for Oenomaus cyanovenata

XML Treatment for Oenomaus druceus

XML Treatment for Oenomaus floreus

XML Treatment for Oenomaus gaia

XML Treatment for Oenomaus geba

XML Treatment for Oenomaus griseus

XML Treatment for Oenomaus isabellae

XML Treatment for Oenomaus jauffreti

XML Treatment for Oenomaus magnus

XML Treatment for Oenomaus melleus

XML Treatment for Oenomaus morroensis

XML Treatment for Oenomaus nigra

XML Treatment for Oenomaus ortygnus

XML Treatment for Oenomaus poirieri

XML Treatment for Oenomaus taua
